# Hippotherapy for Children with Autism Spectrum Disorder: Executive Function and Electrophysiological Outcomes

**DOI:** 10.3390/brainsci16040413

**Published:** 2026-04-14

**Authors:** Zahra Mansourjozan, Sepehr Foroughi, Amin Hekmatmanesh, Mohammad Mahdi Amini, Hamidreza Taheri Torbati

**Affiliations:** 1 Laboratory of Intelligent Machines, LUT University, 53850 Lappeenranta, Finland; z.mansur.j@gmail.com (Z.M.); sepehr.for@gmail.com (S.F.); 2 Department of Physical Education and Sport Sciences, Faculty of Humanities, Islamic Azad University, Mashhad 9187147578, Iran; mmamini33@yahoo.com; 3 Department of Motor Behavior, Ferdowsi University of Mashhad, Mashhad 9177948974, Iran; hamidtaheri@um.ac.ir

**Keywords:** hippotherapy, autism spectrum disorder, executive function, resting-state EEG, phase lag index, partial transfer entropy

## Abstract

**Highlights:**

**What are the main findings?**

- A standardized 12-session hippotherapy program produced large, significant improvements across multiple executive function measures in children with ASD compared with waitlist controls (adjusted *p* < 0.001; partial η^2^ = 0.47–0.86).
- Hippotherapy was associated with relative stabilization of resting-state EEG spectral and connectivity dynamics—particularly in frontal delta, theta, alpha, and beta bands—although EEG changes did not map directly onto individual behavioral gains.

**What are the implications of the main findings?**

- The results support hippotherapy as a promising sensorimotor intervention to enhance executive function in children with ASD and justify larger randomized trials with active control conditions.
- Future research should include task-evoked electrophysiological measures and extended longitudinal follow-up to clarify mechanisms and the durability of effects.

**Abstract:**

**Background:** Hippotherapy, a sensorimotor-rich intervention proposed for children with Autism Spectrum Disorder (ASD), is suggested to influence executive function (EF). However, the underlying electrophysiological mechanisms, particularly changes observed in resting-state Electroencephalography (EEG), remain underexplored. **Methods:** A total of forty-eight children with ASD, aged 9–12 years, participated in this quasi-experimental, non-randomized pre-test–post-test study. Participants were assigned to either a standardized 12-session hippotherapy program (*n* = 24) or a waitlist Control group (*n* = 24). EF was evaluated pre- and post-intervention using validated measures: the Wisconsin Card Sorting Test, Stroop Color–Word Test, Corsi Block-Tapping Task, and Tower of London. Resting-state EEG data (19 channels, 250 Hz) were recorded before and after the intervention and analyzed for spectral power, pairwise Pearson correlation, phase-based functional connectivity using the Phase Lag Index (PLI), and directed effective connectivity using Phase Transfer Entropy (PTE). EEG effects were tested with linear mixed models in MATLAB (fitlme), with the measured values in each ROI as the dependent variable, group and time as fixed effects, and SubjectID included as a random intercept; EF outcomes were analyzed with ANCOVA/MANCOVA, adjusting post-test scores for baseline. The assumptions of homogeneity of slopes, Levene’s test, and the Shapiro–Wilk test were examined, and the Holm–Bonferroni correction together with partial η^2^ effect sizes were reported. **Results:** Following baseline adjustment, the hippotherapy group showed substantial and statistically significant improvements across all EF measures compared with controls partial η^2^ range = 0.473–0.855; all adjusted *p* < 0.001; e.g., Stroop Incongruent Reaction Time (*F*(1,45) = 265.80, *p* < 0.001, *ηp*^2^ = 0.855). EEG analyses revealed localized Group × Time interaction effects involving frontal delta power as well as selected alpha-, theta-, and beta-band connectivity measures within frontally anchored networks. In addition to these focal interaction effects, the hippotherapy group exhibited a narrower distribution of pre–post EEG changes across spectral power and connectivity metrics compared with controls, indicating greater temporal consistency in resting-state electrophysiological dynamics across sessions. Because group allocation was non-random (based on scheduling feasibility and parental preference), results should be interpreted as associations rather than causal effects. While the hippotherapy group exhibited significant EF improvements and relative stabilization in EEG spectral and connectivity metrics, particularly in frontal delta/theta/alpha/beta bands, a direct mapping between individual EEG changes and behavioral gains was not observed. **Conclusions:** A standardized 12-session hippotherapy program was associated with substantial improvements in EF and with relative stabilization of resting-state electrophysiological dynamics in children with ASD. However, the direct mechanistic link between these EEG and behavioral changes warrants further investigation. Larger randomized trials employing active control conditions, task-evoked electrophysiological measures, and extended longitudinal follow-up are needed to confirm efficacy, clarify mechanisms, and establish the durability of effects.

## 1. Introduction

Autism Spectrum Disorder (ASD) is a multifaceted neurodevelopmental condition characterized primarily by persistent difficulties in social communication and interaction, alongside restricted and repetitive behaviors, with a wide range of symptom severity and functional variability across individuals [1]. Beyond these hallmark features, many children with ASD experience significant executive function (EF) impairments, including deficits in working memory, inhibitory control, planning, and cognitive flexibility. These EF challenges undermine adaptive behaviors, academic achievement, and social participation [2].

Neuroimaging and electrophysiological research have consistently revealed atypical development and impaired connectivity within large-scale brain networks, particularly those involving the frontoparietal cortex and cerebellum [3,4]. Such disruption of long-range neural communication is thought to contribute to goal-directed behavior and cognitive control, core difficulties underpinning ASD symptomatology [5]. These altered neural dynamics may represent the neural substrate for the executive dysfunctions and adaptive challenges documented in affected individuals.

Conventional behavioral and pharmacological interventions rarely yield sustained EF improvements in ASD treatment, underscoring the need for novel, multisensory approaches. Hippotherapy is an equine-assisted therapy (EAT) that leverages the horse’s movement to provide multifaceted sensory input [6]. It is important to note that while often associated with horseback riding, hippotherapy encompasses a spectrum of interactions with the horse. These can range from activities like grooming and leading the horse to therapeutic horseback riding itself, all guided by a physical therapist, occupational therapist, or speech-language pathologist who has completed the robust training and credentialing process [7]. This dynamic interaction utilizes the horse’s rhythmic gait to deliver continuous vestibular, proprioceptive, and tactile stimulation, which is crucial for engaging postural control, balance, and adaptive responses. The multisensory nature of hippotherapy, extending beyond mere physical exercise, offers a unique avenue for potentially enhancing cognitive functions such as attentional flexibility and EFs, particularly relevant for individuals with ASD [7,8]. This therapist-guided approach harnesses the horse’s rhythmic, three-dimensional gait to provide continuous proprioceptive, vestibular, and tactile stimulation [9]. Such dynamic sensory engagement is theorized to enhance attentional flexibility, inhibitory control, and goal-oriented planning, as demonstrated in other populations [10,11]. Specifically, the constant need to process and adapt to the horse’s movement demands active cognitive engagement. This involves filtering irrelevant sensory information, shifting focus as needed, inhibiting impulsive reactions, and maintaining a consistent goal (e.g., staying balanced, following instructions). This continuous mental effort acts like a “cognitive workout,” strengthening the neural pathways that support EFs [7,12,13].

Accumulating evidence supports hippotherapy’s potential in ASD. Randomized controlled trials report reductions in irritability and hyperactivity [12], gains in postural control and EF following single and multi-session programs [13,14,15], and broader improvements in social communication and stereotyped behaviors [7,16]. The potential of hippotherapy extends to autonomic regulation, as suggested by recent findings [8]. Meta-analytic findings indicate faster problem-solving and shorter reaction times compared with control activities [17], suggesting benefits beyond those attributable to general physical exercise.

Despite the promising behavioral outcomes reported in recent studies, our understanding of the neural mechanisms underlying hippotherapy’s effects in children with ASD remains limited [12,17]. Electroencephalography (EEG) offers a sensitive, non-invasive approach to monitoring brain activity related to EF [18]. For instance, increased alpha-band power has been associated with enhanced cortical inhibition and attentional gating [19], while beta-band activity reflects sensorimotor integration and higher-order executive processing [20]. Advanced measures, such as Phase Transfer Entropy (PTE), enable the quantification of directional connectivity between frontal and posterior regions, which are crucial pathways for EF. However, interpreting EEG data in ASD is complex due to heterogeneous baseline rhythms and typical underconnectivity patterns reported in the literature [18,21]. As a result, pre- and post-intervention designs are essential for elucidating the neural impact of hippotherapy.

To address this gap, we enrolled 48 children with ASD (ages 9–12 years) and compared a standardized 12-session hippotherapy program to a waitlist Control group. EF was assessed with validated measures targeting cognitive flexibility, response inhibition, working memory, and planning. Resting-state EEG was recorded pre- and post-intervention to quantify band-limited spectral power, phase-based functional connectivity, and directed effective connectivity within the alpha, beta, and gamma bands.

This study therefore tested two hypotheses: first, that a short course of hippotherapy yields measurable improvements in core EFs; and second, that any behavioral gains may be accompanied by or associated with reorganization or stabilization of large-scale functional brain networks. Investigating these potential neural mechanisms by examining the relationship between behavioral changes and EEG alterations will improve our understanding of how sensorimotor-rich interventions influence the neurocognitive profile of ASD and will inform the design of neuroscience-informed therapeutic approaches.

## 2. Materials and Methods

We employed a quasi-experimental pre-test–post-test design with a non-randomized waitlist Control group to assess the effects of a 12-session hippotherapy program on EF and EEG indices in children with ASD. The study initially involved 48 children diagnosed with ASD, all of whom were considered higher-functioning based on their diagnostic profiles and pre-study assessments. Although groups were matched in age and baseline characteristics, assignment was determined by scheduling feasibility and parental choice rather than randomization. This introduces potential selection bias and limits the ability to draw strong causal inferences. As such, the observed effects are associated findings within a non-randomized framework, limiting direct causal claims.

### 2.1. Ethics and Documentation

The study protocol and a detailed statistical analysis plan were prepared prior to data collection. The full protocol is available in the Supplementary File (Table S1). At the time the study began, local guidance did not require registration of non-randomized behavioral studies. The project was approved by the University Ethics Committee and the Faculty Research Council of the University of Tabriz and was conducted in accordance with national ethical standards in Iran. Written informed consent was obtained from parents, and assent was provided by each child.

### 2.2. Allocation and Efforts to Minimize Bias

Participants were allocated to the Experimental (hippotherapy) or waitlist Control group using a non-randomized procedure driven by scheduling feasibility and parental preference. To mitigate potential baseline imbalances, groups were balanced on age, sex, and baseline executive function measures, including WCST (two indices), Corsi span, Tower of London, and Stroop (two indices). Baseline characteristics of the participants and the standardized mean differences (Cohen’s d) for all covariates are presented in Supplementary Table S4. In addition, we implemented the following methodological safeguards: (a) enrolled participants were balanced at baseline for age and autism severity; (b) standardized mean differences were calculated for all baseline covariates; and (c) primary analyses were adjusted using analysis of covariance (ANCOVA) with pre-test scores entered as covariates. Supplementary longitudinal models were also estimated to assess the robustness of findings. Furthermore, to maximize participant retention and engagement, several supportive measures were implemented. Sessions were offered with significant flexibility in scheduling to accommodate family logistics. All hippotherapy services, including transportation costs for parents and children, were fully covered to remove financial barriers. The inherent appeal of interacting with horses served as a strong motivational factor for many participants. While initial apprehension regarding horse-related activities was noted in a small subset of families, this was rapidly alleviated through the consistent presence of our multidisciplinary support team. This team, comprising a child psychiatrist, psychologist, hippotherapy specialist, and three equine assistants, provided a secure and reassuring environment, contributing to the high retention rate observed throughout the study. These combined factors likely played a role in the absence of dropouts from the final sample.

### 2.3. Data Handling and Statistical Comparisons

Data were screened for completeness prior to analysis; no missing data were identified among the final sample (*n* = 48), and all analyses were conducted on complete cases. Baseline comparability between the Experimental and Control groups was evaluated for demographic variables (age, gender) and pre-test measures of EF using independent-samples Welch’s *t*-tests and standardized mean differences (Cohen’s *d* for continuous variables, Cramer’s *V* for categorical). No statistically significant differences were observed (*p* > 0.05 for all comparisons). Effect sizes indicated good baseline equivalence: Age (*d* = 0.422), Gender (Cramer’s *V* = 0.042), WCST categories (*d* = −0.205), WCST perseverative errors (*d* = 0.023), Corsi span (d = 0.057), Tower of London moves (*d* = 0.004), Stroop Congruent RT (*d* = 0.195; negligible), and Stroop Incongruent RT (*d* = −0.161; small). While formal quantitative comparisons for IQ and autism symptom severity were not conducted due to data collection constraints, the homogeneity of the sample regarding these variables was ensured through stringent eligibility criteria, including a minimum full-scale IQ of 70 and a confirmed ASD diagnosis (see Section 2.4). Given the non-random assignment of participants, all primary outcome analyses adjusted for baseline scores of key variables to enable transparent interpretation of causal inferences.

### 2.4. Eligibility and Exclusion Criteria

Participants were meticulously screened to ensure suitability for the study, aligning with our objective to investigate the potential benefits of hippotherapy on specific aspects of EF in children with ASD.

#### 2.4.1. Inclusion Criteria

To form a homogeneous and representative sample for our targeted investigation, eligible participants met the following criteria:

- A confirmed diagnosis of ASD, based on DSM-5 criteria, established by experienced clinicians.
- Participants aged 9 to 12 years were included. This age range was selected to target a developmental period where core EFs are actively maturing and responsive to intervention, yet sufficiently developed for reliable assessment and preceding the significant neurobiological changes in puberty. This ensures developmental stability, minimizes confounds for EEG recording (where compliance is also higher), and allows for consistent engagement in structured activities like hippotherapy, thereby enhancing the validity and interpretability of the study’s findings.
- A full-scale IQ above 70, coupled with the absence of co-occurring intellectual disability. This criterion was crucial for ensuring that participants could meaningfully engage with the cognitive assessments and intervention tasks, and allowed us to focus on the specific EF challenges associated with ASD in typically developing cognitive ranges.
- No prior exposure to EATs. This was essential to isolate the effects of the novel intervention and avoid confounding variables related to previous therapeutic experiences.

This comprehensive screening process was conducted collaboratively by a child psychiatrist and a clinical psychologist specializing in ASD. Their assessment included a thorough review of each child’s diagnostic profile, a detailed evaluation of their functional abilities, and a careful consideration of any contraindications for physical activity or engagement with animals.

#### 2.4.2. Exclusion Criteria

The following exclusion criteria were implemented to uphold participant safety and ensure the scientific integrity and interpretability of the study’s outcomes:

- History of self-injurious behavior or suicidal ideation: To prioritize and ensure the absolute safety of all participants throughout the study duration.
- Aggression toward animals: To maintain a safe and positive therapeutic environment for the participant, the therapy provider, and the equine partner.
- Major psychiatric or medical conditions (e.g., epilepsy, uncontrolled asthma): To mitigate potential health risks associated with physical activity and the therapeutic setting, and to prevent exacerbation of pre-existing conditions that could impact participation or outcome interpretation.
- Sensory or motor impairments that could interfere with assessments: To guarantee the reliability and validity of the cognitive and behavioral outcome measures, ensuring that observed changes are attributable to the intervention and not compromised by assessment limitations.

#### 2.4.3. Medication and Concurrent Support Status

Prior to study enrollment, a comprehensive assessment of each participant’s medication regimen and existing therapeutic support was conducted by the on-site psychiatrist and psychologist to ensure they met the inclusion criteria. Participants’ medication status was monitored for stability throughout the intervention period, with baseline prescriptions documented by the study psychiatrist and parents instructed to promptly report any changes in medication use (including dose adjustments, discontinuation, missed doses, or forgetfulness) to the research team. Crucially, all participants maintained their pre-existing, stable medication schedules without initiation of new psychotropic medications or significant dosage adjustments. Similarly, any concurrent educational supports or extracurricular therapies the participants were engaged in prior to the study remained unchanged during the 12-session intervention period. Furthermore, participants’ engagement in regular physical activities outside of the study protocol remained consistent throughout the intervention period. This careful management of concurrent treatments and medication stability was essential to isolate the potential effects of the hippotherapy intervention itself and avoid confounding outcomes by the introduction or alteration of other therapeutic modalities or pharmacological agents.

### 2.5. Equine Selection and Matching

The efficacy and safety of hippotherapy are significantly influenced by the selection and suitability of the equine partners. For this study, a cohort of five therapy horses was utilized, all of whom were geldings aged between 7 and 15 years. These horses were carefully selected based on their temperament and physical soundness, undergoing a prescreening process conducted by the study’s equine manager and the treating therapist. Prior to their inclusion, each horse received a veterinary assessment to ensure they were fit for therapeutic work. Each child was assigned their primary equine partner for the duration of the study to minimize variability in sensory experience and foster a consistent therapeutic relationship, with any necessary substitutions meticulously documented. Further details regarding the specific characteristics of each therapy horse are provided in Supplementary Table S2.

### 2.6. Risk Assessment, Safety Protocols, and Staff Qualifications

Ensuring the safety of participants and staff, alongside the welfare of the therapy horses, was paramount throughout the intervention. A comprehensive risk assessment was conducted prior to the commencement of the study. The safety protocol encompassed several layers, as follows.

#### 2.6.1. Equine Welfare and Safety Checks

A detailed checklist (Supplementary File S1) was employed to assess both equine welfare and session safety before, during, and after each therapy session. This included inspecting the arena for appropriate footing and environmental conditions (e.g., noise levels, temperature). Horses exhibiting signs of stress, such as ear pinning, tail swishing, or increased muscle tension, were immediately removed from the session or substituted.

#### 2.6.2. Supervision Ratios

During mounted activities, each horse–participant pair was supervised by a dedicated lead handler and two credentialed professional sidewalkers. The lead therapist was supported by three credentialed professional assistants, maintaining a therapist-to-child ratio of approximately 1:2. This high level of supervision ensures immediate response to any potential safety concerns.

#### 2.6.3. Staff Training and Credentials

The intervention was delivered by a lead therapist with a doctoral-level specialization in motor behavior and over 10 years of clinical experience. This therapist is also a certified riding instructor with more than 15 years of professional experience. Crucially, they have completed nationally accredited training programs in hippotherapy and EAT, comparable to American Hippotherapy Association (AHA) Level I-II standards. Furthermore, the therapist holds current certifications in Cardiopulmonary Resuscitation (CPR), First Aid, and Child Safeguarding. All staff underwent annual AHA-mandated safety and crisis-response training, as detailed in Supplementary Table S3. Clinical oversight was consistently provided through weekly consultations with a multidisciplinary team, including a child psychiatrist, a physical therapist, and an occupational therapist.

#### 2.6.4. Absence of Adverse Events

Throughout the 12-session program, no adverse events were reported among participants or staff.

### 2.7. Intervention Protocol: The 12-Session Hippotherapy Program

The intervention group engaged in a structured six-week hippotherapy program comprising 12 sessions, delivered twice weekly. Each session was designed to be 25–35 min in duration, aligning with established best-practice recommendations for providing targeted sensorimotor input while mitigating participant fatigue and maintaining engagement [6,12]. The protocol was manualized to ensure consistency and fidelity, as detailed in Supplementary Table S1. Detailed procedures for monitoring intervention fidelity are described in Supplementary File S2.

The intervention comprised two integrated phases: a foundational off-horse phase (Sessions 1–5) and a mounted hippotherapy phase (Sessions 6–12). The off-horse phase was crucial for establishing a secure therapeutic relationship, enhancing sensory awareness, and preparing the patient’s sensory and motor systems for the demands of mounted work, thereby ensuring optimal readiness for the core sensorimotor tasks. This preparatory phase is grounded in principles of therapeutic engagement and patient readiness, critical for maximizing the efficacy of subsequent direct hippotherapy [6,7,12].

Each session followed a standardized structure:

Orientation and Safety Review (3–5 min): A brief period to reorient participants to the environment and review safety procedures.

Structured Sensory–Motor or Social-Emotional Tasks (20–25 min): This core component involved activities specifically designed to provide controlled and graded vestibular and proprioceptive input through the horse’s natural three-dimensional gait. Examples included mounted postural challenges, graded changes in speed executed by the horse, and navigation tasks within the arena.

Cool-down/Reflection Phase (3–5 min): A brief period for participants to wind down and reflect on their experience.

Prior to the mounted activities, all participants engaged in supervised horse-care activities, which included grooming the horse (brushing, combing mane and tail) and brief, controlled hand-feeding. These ground-based interactions were implemented to foster participant comfort and mitigate stress, aligning with AHA standards and protocols [6,12]. Intervention fidelity was rigorously monitored through therapist checklists and standardized rubrics, with supervisory observation of approximately 10% of sessions. Weekly team reviews further supported adherence to the manualized protocol. Any necessary individual adaptations, such as shortening a riding segment due to participant fatigue or sensory sensitivity, were recorded but did not alter the core structure of the session.

### 2.8. Control Group Protocol

To control for non-specific factors such as therapist attention and time out of routine, a time- and attention-matched protocol was implemented for the Control group. Control sessions were designed to provide comparable exposure to the stable environment and therapist interaction, while deliberately minimizing direct equine-mediated sensorimotor stimulation. Activities for the control group included the following:

Brief, Passive Equine Exposure: Participants engaged in short, supervised activities such as gentle grooming or observation of the horses, not exceeding 5 min per session. This exposure was carefully managed to limit active sensorimotor input from the horses.

Ground-Based Therapeutic Activities: Therapist-led engagement in play, conversation, or other non-mounted sensory activities was conducted in an adjacent, quiet area of the stable.

These activities were matched in duration (e.g., 45 min per session) and the level of general attention provided by the therapist to the intervention sessions. Participants in both groups attended all 12 scheduled sessions, resulting in a 100% attendance rate and no dropouts, ensuring comparable overall exposure duration and therapeutic engagement across groups.

### 2.9. Fidelity Monitoring

Intervention fidelity was a fundamental aspect of this study, ensuring that the hippotherapy protocol was implemented consistently and precisely as designed. Fidelity procedures were maintained through several monitoring strategies (see Supplementary File S2).

Manualized Session Plan: The intervention followed a structured, pre-defined manualized session plan (Supplementary Table S1) that detailed specific therapeutic activities, objectives, and safety procedures for each type of session.

Therapist Checklists and Standardized Rubrics: The primary therapist systematically completed checklists and rubrics after each session to document adherence to the protocol, including the types of activities performed, duration, and any adaptations made.

Supervisory Observation: An independent supervisor observed approximately 10% of the intervention sessions to provide an objective assessment of fidelity and provide feedback.

Weekly Team Reviews: Regular team meetings were held to discuss session progress, address any challenges, and reinforce adherence to the manualized protocol.

This multifaceted approach to fidelity monitoring ensured that the observed effects could be reliably attributed to the hippotherapy intervention as designed.

### 2.10. Assessment Blinding and Bias Mitigation

All cognitive assessments (Wisconsin Card Sorting Test, Corsi Block-Tapping Task, Stroop Color–Word Test, and Tower of London) were administered by trained examiners who were blinded to participants’ group assignments. Examiners followed standardized administration protocols and completed structured session checklists to ensure procedural consistency. Cognitive testing took place within one week before the first intervention session and within one week after the final (12th) session. To mitigate potential order effects, the sequence of cognitive assessments administered to each participant was systematically counterbalanced using a pre-defined randomization protocol, ensuring that task order did not systematically influence performance.

Standardized Administration with Necessary Adjustments for Children with ASD:

To ensure valid and reliable performance, particularly for children with high-functioning ASD in our sample, cognitive assessments were administered following standardized protocols. While the core demands of the tasks remained unaltered, brief, pre-defined adjustments were permitted to facilitate comprehension and minimize potential anxiety or fatigue. These adjustments, applied on an individual basis when deemed necessary by the trained examiners, included:

- Repetition of instructions: Instructions were repeated verbatim if a participant showed signs of not fully understanding the task.
- Extended time for task comprehension: A modest extension of time was provided solely for understanding the task instructions or the practice trial, not for the cognitive task performance itself.
- Brief task demonstrations: Clarifying task demonstrations were used to illustrate the response format.

These accommodations, detailed in Table S5 of the Supplementary Materials, were applied judiciously and consistently across both intervention and Control groups, documented meticulously by examiners via structured session checklists. The aim was to optimize the conditions for participants to demonstrate their true cognitive abilities without altering the fundamental nature or cognitive load of the assessments. All uses of these accommodations, along with details of any partial task administrations, are documented in the Supplementary Materials. No participant was excluded from the cognitive battery due to difficulties in task completion.

To ensure the independence of our cognitive assessors and rigorously mitigate expectancy bias, we implemented a stringent blinding protocol. Assessors were fully unaware of participant group allocation (hippotherapy intervention vs. Control). Participants were identified solely by unique, anonymized IDs, preventing any group identification. Crucially, assessors maintained complete separation from therapists and participants, having no interaction during the intervention period or presence in treatment sessions. This ensured that data collection and analysis proceeded without any potential information leakage. While we did not formally poll assessors about any potential guesses regarding group assignments, the standardized nature of the cognitive assessments, which are independent of intervention specifics, combined with the strict separation from the treatment process, leads us to conclude that the risk of a compromised blind or expectancy bias influencing our results is minimal.

Assessors provided neutral prompts or procedural clarifications when appropriate but did not give performance-related feedback or strategic guidance. This approach, which involved blinded, well-trained assessors using standardized scripts and transparent reporting of minimal, necessary accommodations tailored for children with ASD, was chosen to preserve methodological rigor.

### 2.11. Cognitive Measures

EF were measured using a battery of standardized computerized tasks with established psychometric properties.

#### 2.11.1. Wisconsin Card Sorting Test (WCST)

The WCST assessed cognitive flexibility and set shifting. Modified versions of the WCST show excellent split-half reliability (coefficients > 0.90) for primary indices such as categories achieved and perseverative errors; traditional forms report moderate test–re-test reliability (*r* = 0.30–0.70) depending on sample and task complexity [22]. Higher category scores indicate greater flexibility; higher perseverative error counts indicate poorer performance. In our scoring conventions, perseverative errors are in the range of 0–128 and categories achieved a range of 0–6.

#### 2.11.2. Corsi Block-Tapping Test

The Corsi task measured visuospatial working memory by requiring participants to reproduce sequences of tapped blocks. Both traditional and computerized versions (e.g., eCorsi) demonstrate high test–re-test reliability (*r* ≈ 0.81–0.89) [23]. Higher scores reflect stronger visuospatial working memory. Standard sequence lengths typically span two to nine blocks.

#### 2.11.3. Stroop Color–Word Test

The Stroop test indexed response inhibition and selective attention. It has moderate internal consistency (Cronbach’s α ≈ 0.74 for interference scores) and is associated with conflict monitoring and working memory processes [24]. Greater interference scores indicate weaker inhibitory control. Reaction times (RTs) are reported in milliseconds; analyses focused on the incongruent condition, where RTs in this sample were in the approximate range of 650–1010 ms.

#### 2.11.4. Tower of London Test

The Tower of London evaluated planning and problem-solving. It demonstrates acceptable internal consistency (Cronbach’s α ≈ 0.79) with normative performance varying by age and other demographics [25]. Higher total scores and shorter completion times denote better planning efficiency. In the standard 12-item version, total moves typically range from 30 to 56.

### 2.12. EEG Data Acquisition

EEG is a non-invasive neuroimaging technique that records electrical activity generated by neuronal firing in the brain [26]. The signals are captured through electrodes placed on the scalp surface, allowing researchers to examine functional brain responses and neurocognitive processes. Due to its excellent temporal resolution, EEG can detect neural activity occurring within milliseconds [27,28]. By analyzing the recorded EEG signals, it is possible to extract quantitative features associated with brain connectivity and underlying neural dynamics.

**Figure 1:**
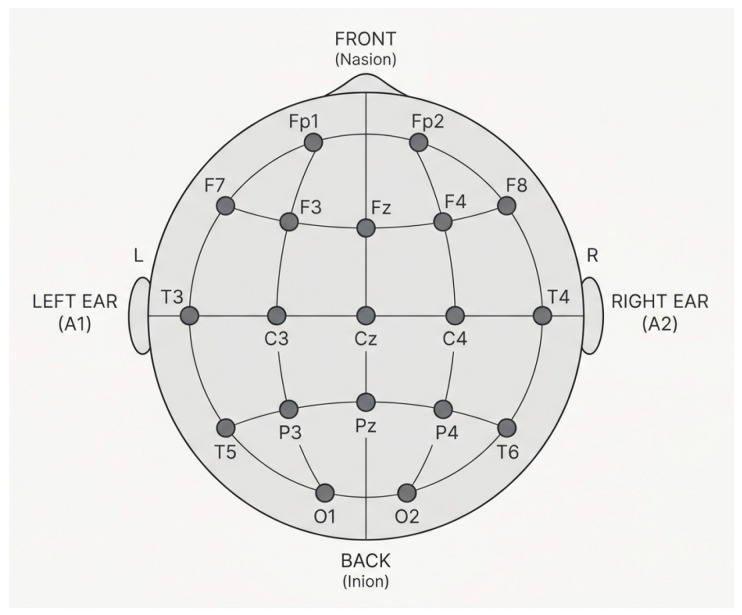
The 19-channel electrode locations in the International 10–20 EEG System.

EEG signals were recorded from 19 channels in 48 participants, both before and after the intervention, at a sampling rate of 250 Hz (see Figure 1).

### 2.13. EEG Signal Preprocessing

We used a moderate preprocessing pipeline to match each downstream measures’ sensitivities. At first, we used an FIR 0.5–80 Hz bandpass filter of order 2500 along with an FIR 50 Hz notch filter of order 1500. The filters were applied to the continuous EEG signals using EEGLAB’s zero-phase FIR filtering (pop_eegfiltnew). Because long FIR filters can introduce transient distortions at the boundaries of the filtered signal, the first 10 s of each recording were discarded to remove potential filter transients. The remaining continuous data were then used for subsequent preprocessing and analysis. For the sake of phase-based measures, we prioritized preservation of the phase. Thus, the EEGLAB Clean Rawdata plugin, implementing Artifact Subspace Reconstruction (ASR) [29], was applied to remove bad data segments and corrupted channels, along with additional removal of bad data periods. Flat channels (lasting > 5 s), high-noise channels (standard deviation > 4), and poorly correlated channels (correlation < 0.8) were excluded. ASR was applied using a 0.5 s window with a standard deviation cutoff of 10. Additional bad data periods were rejected based on RMS > 7 SD and had >5 corrupted channels simultaneously, and all rejected data were visually checked. Independent Component Analysis (ICA) was then used to remove the eye noise and muscle noises from the datasets. This process was held using EEGLAB ICA toolbox runica [30,31]. The number of independent components was equal to the number of remaining EEG channels after preprocessing, which varied between participants (approximately 14–19 channels). Components classified as non-brain with a probability greater than 90% by ICLabel as eye or muscle noise were marked for rejection and visually inspected to confirm their classification before removal [32]. ICA was applied as an auxiliary step to identify potential residual ocular or muscle artifacts after ASR cleaning, rather than as a primary denoising method. The conservative ICLabel probability threshold (>90%) was deliberately chosen to minimize the risk of removing neural signal components and to avoid potential phase distortion. As a result, in several participants, no components met the rejection criteria, and ICA did not necessarily lead to component removal in all datasets.

The length of continuous EEG data used for ICA fitting was sufficient in all participants, with a minimum duration of 49 s and a mean duration of 216 s across both groups. Because ICA was not used for source localization and did not play a dominant role in signal reconstruction, formal ICA fitting quality metrics (e.g., residual variance) were not computed.

**Figure 2:**
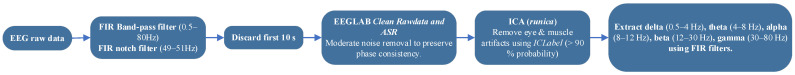
Flowchart of EEG signal preprocessing.

After the data were cleaned, the five sub-frequency bands, i.e., delta (0.5–4 Hz), theta (4–8 Hz), alpha (8–12 Hz), beta (12–30 Hz), and gamma (30–80 Hz), were extracted from the data using FIR filters of orders 2000 for the delta and theta bands and 1000 for the alpha, beta, and gamma bands (Figure 2).

For subsequent analyses—including spectral power, correlation, PLI, and PTE—the band-limited signals were segmented into 8 s windows with 50% overlap. This window length was selected as a practical compromise between temporal resolution and reliable phase estimation. At the lowest analyzed frequency range (delta), an 8 s segment contains roughly 15 oscillatory cycles, which is generally considered sufficient for stable estimation of phase-based connectivity measures [33,34], while still allowing for consistent analysis across frequency bands.

The first 10 s of each recording had already been removed after the broadband and notch filtering step to eliminate potential filter transients, and no additional trimming was applied after the band-specific filtering. Filtering was performed using EEGLAB’s pop_eegfiltnew function, which implements zero-phase FIR filtering with internal signal padding to reduce boundary distortions [35]. Any remaining edge effects were therefore expected to be minimal and were further reduced by averaging the measures across multiple overlapping windows.

#### 2.13.1. Power Analysis

Analyzing the power of specific EEG frequency bands can give important information about the brain’s functional state. This method splits the EEG into different frequency components and measures the power in each band, which is called the Power Spectral Density (PSD). In ASD, previous studies have often found characteristic differences in EEG band power [36,37].

PSD shows how the power of a signal is distributed over frequency and is useful for studying non-stationary and oscillatory behavior in EEG. To estimate PSD, a common method is the Discrete Fourier Transform (DFT) [38]. For a discrete-time signal x[n], the DFT converts it into the frequency domain as:

$$X\left[k\right]=\sum_{n=0}^{N-1}x\left[n\right].e^{-j2\pi kn/N}$$

where N is the number of points and *k* indexes the frequency components. A simple PSD estimate, called the periodogram, is obtained by squaring the magnitude of the DFT:

$$P\left[k\right]=\frac{1}{N}{\left|X\left[k\right]\right|}^{2}$$

This gives the power at each frequency bin [38]. Because single-period estimates can be noisy, more stable PSD estimates are made using Welch’s method. Welch’s method divides the signal into overlapping segments, applies a window function (like Hamming), and computes the PSD for each segment and averages them across all segments. We calculated PSD in 8 s window lengths and 50% overlaps.

#### 2.13.2. Connectivity Estimation

To assess whether the intervention led to any changes in the participants’ brain circuits and connectivity, we calculated PTE, Phase Lag Index (PLI), and pairwise Pearson correlation across the EEG channels, which measures effective connectivity.

#### 2.13.3. Correlation

Amplitude-based connectivity was calculated as pairwise Pearson correlation in 8 s windows with 50% overlap across channels for each subject. Pearson correlation is a method that measures instantaneous correlation across two vectors. The formula for Pearson correlation is:

$$r_{X,y}=\;\frac{\sum_{i=1}^{n}\left(x_{i}-\;\bar{x})(y_{i}-\;\bar{y}\right)}{\sqrt{\sum_{i=1}^{n}{\left(x_{i}-\;\bar{x}\right)}^{2}}\;\sqrt{\sum_{i=1}^{n}{\left(y_{i}-\;\bar{y}\right)}^{2}}\;}$$

where n is the sample size, and $x_{i}$, $y_{i}$ are the individual sample points indexed with i, $\bar{x}=\frac{1}{n}\sum_{i=1}^{n}x_{i}$ (the sample mean) and analogously for $\bar{y}$. The calculations were held in MATLAB (R2024a) corr function [39].

#### 2.13.4. Phase Transfer Entropy (PTE)

PTE is a phase-based refinement of Transfer Entropy (TE), an information-theoretic, model-free, nonparametric estimator of directed (time-asymmetric) information transfer between time series [40]. Because TE makes no assumptions about the form of interactions, it can detect both linear and nonlinear dependencies, which is advantageous for analyzing complex physiological signals. By computing TE on instantaneous phase time series, PTE reduces sensitivity to common electrophysiological confounds (e.g., noise, linear mixing, and volume conduction) and is computationally efficient for oscillatory EEG/MEG data [41]. Directed variants (dPTE) have been applied in pediatric samples [42]; although direct dPTE studies in children with autism are still limited, related phase-based entropy measures, including normalized PTE and Phase Lag Entropy, have revealed altered directed information flow and greater connectivity diversity in ASD cohorts [3,43].

For a system with two variables X and Y, let $P_{Y}$ be the probability of Y and $P_{X,Y}$ be the joint probability of X and Y. The joint entropy is:

$$H\left(x,y\right)=-\sum_{x\epsilon X}\sum_{y\epsilon Y}P_{X,Y}(x,y)\log P_{X,Y}(x,y)$$

The conditional entropy is:

$$H\left(X|Y\right)=H\left(X,Y\right)-H\left(X\right)$$

This means $H\left(X|Y\right)$ is the uncertainty left in Y after knowing X. Then *TE*, which shows directional flow, is:

$$TE\left(X\to Y\right)=H(Y_{F}|Y^{P})-H(Y^{F}|X^{P}Y^{P})$$

Here, $Y_{F}$ is future Y values shifted by a time lag, and $X^{P}$ and $Y^{P}$ are past values of X and Y [40].

PTE constructs TE from the phase time series of neural signals and is well suited for estimating directed, phase-based connectivity [41]. In autism research, for example, Wang et al. (2022) applied normalized PTE (nPTE) to resting-state MEG recordings from children and reported significant alterations in gamma-band network activity and directed information flow in children with ASD compared with typically developing peers [3]. Other work has applied phase-based entropy measures such as Phase Lag Entropy to characterize greater connectivity diversity and altered dynamic phase relationships in pediatric ASD cohorts [3]. Finally, recent empirical and review studies indicate that entropy- and TE-based indices provide complementary and valuable insights into effective (directed) connectivity abnormalities in ASD and may help identify EEG biomarkers [4].

We calculated PTE between EEG channels using the PhaseTE_MF toolbox, with parameters ‘delay’ and ‘binsize’ set to ‘default’ and ‘otnes’, respectively. Signals were cut into 8 s windows with 50% overlap. PTE was calculated for each window and averaged [44].

#### 2.13.5. Phase Lag Index (PLI)

PLI is a measure of functional connectivity proposed by Stam et al. to address the problem of volume conduction and active reference electrodes in EEG signals [45]. Specifically, PLI quantifies consistent non-zero-lag phase relationships between two EEG signals, i.e., phase coupling. It ignores zero-lag coupling, which is often contaminated by volume conduction or common reference, so it focuses on genuine functional interactions rather than spurious correlations. Its values range from 0 (no consistent phase relationship) to 1 (perfectly consistent phase lead/lag) [45].

Mathematically PLI can be expressed as

$$PLI=\left|\langle sign\left[\Delta \varphi (t_{k})\right]\rangle \right|$$

where $\Delta \varphi (t_{k})$ denotes a time series of phase differences, |·| denotes the absolute value, 〈·〉 denotes the arithmetic mean, and sign(·) is the sign function.

From the artifact-cleaned EEG recordings, a continuous 32 s segment was selected to calculate phase-based connectivity (PLI). Importantly, this segment was not necessarily taken from the first 32 s of the original recording, nor was it fixed to the same temporal position across participants. Instead, segments were selected from portions of the preprocessed data that were free of visible artifacts after the cleaning procedures (including ASR and ICA). As a result, the exact temporal location of the selected segment could vary across participants depending on data quality, and the final selection can be considered quasi-random within the available artifact-free portions of the recording.

This strategy was adopted because maintaining long periods of minimal movement is challenging in pediatric EEG recordings, and longer continuous segments are more likely to contain residual motion artifacts. Selecting a relatively short continuous portion of clean data therefore increases the likelihood of obtaining stable signals suitable for quantitative analysis. In addition, spectral and connectivity analyses generally assume approximate signal stationarity, which is more likely to hold in shorter EEG segments than in longer recordings that may include changes in vigilance or movement-related activity.

Within the selected segment, PLI was estimated using 8 s windows with 50% overlap, resulting in multiple partially overlapping epochs. Connectivity was calculated for each window and subsequently averaged to obtain a single PLI estimate per participant. Averaging across overlapping epochs reduces the influence of short-term fluctuations and provides a more stable connectivity estimate compared with relying on a single window.

A duration of 32 s was considered sufficient to obtain several overlapping analysis windows while maintaining high data quality. The choice of this segment length was also influenced by practical considerations, as PLI estimation across all channel pairs is computationally demanding. Importantly, the aim of the present study was not to investigate methodological effects related to window length, but rather to compare group-level differences in connectivity measures. The use of multiple overlapping windows within the selected segment therefore provided a balance between computational feasibility and robust estimation of connectivity.

Complex signals were calculated and filtered using the Morlet wavelet method and phase values were estimated. Furthermore, PLIs were calculated in 8 s windows with 50% overlaps.

### 2.14. Statistical Analysis

The statistical analysis is divided into two subsections, EEG data and EF data, as detailed below.

#### 2.14.1. EEG Data

We used linear mixed models to test the significance of our results. Linear mixed models are an extension of simple linear models that include both fixed and random effects. They are especially useful when the data are not independent, such as in our EEG measurements [46]. For each Region Of Interest (ROI), a single representative score was computed by aggregating the values across the channels belonging to that region. The aggregation method was selected according to the statistical properties of each measure. Band-limited spectral power was first log-transformed to reduce the positive skew typical of spectral estimates, after which the mean of log-power values across channels within each ROI was calculated. For connectivity analyses, ROI-to-ROI values were derived from all channel-wise connections between electrodes belonging to the two regions. Pairwise Pearson correlation coefficients were averaged using the mean, whereas PLI and PTE values were aggregated using the median to obtain robust estimates in the presence of skewed or heavy-tailed connectivity distributions. Linear mixed-effects models were then fitted separately for each frequency band and for each ROI (power) or ROI pair (connectivity) using the following model:

Measure ~ Group∗Time+ (1|SubjectID)

where *Measure* denotes the ROI or ROI pair score, *Group* and *Time* are the fixed effects, and *SubjectID* is included as a random intercept to account for repeated measurements within participants. No additional covariates were included in the EEG mixed-effects models. Baseline between-subject variability was modeled using a random intercept for each participant (1|*SubjectID*). The dependent variable represented the measured EEG value, while the fixed effects of Time and the Group × Time interaction captured overall temporal changes and group-specific differences in change over time. In addition, demographic and clinical characteristics such as age, sex, and baseline ASD severity were comparable between groups, as reported in the baseline characteristics table. Therefore, further covariate adjustment was not considered necessary for the primary analyses.

*p*-values for the *Group*, *Time*, and *Group* × *Time* coefficients were adjusted for multiple comparisons using the False Discovery Rate (FDR) procedure separately within each frequency band. All LMM results are provided in the Supplementary Tables (Tables S5–S8). Because the primary aim of the study was to evaluate the intervention effect, the main text reports only *Group* × *Time* interaction effects that survived FDR correction.

#### 2.14.2. Dynamicity of EEG Connectivity Measures

To demonstrate the dynamicity of the brain’s connectivity and power parameters between the two groups, we plotted the probability density function (PDF) of the change values for each measure that showed a significant difference between groups. The histograms of these PDFs, along with their corresponding Kernel Density Estimations (KDEs), were plotted.

#### 2.14.3. Executive Function (EF) Data

For the analysis of EF data, we used Python (version 3.13.5) along with essential libraries including Pandas (2.3.1) for data management, NumPy (2.3.5) and SciPy (1.16.3) for statistical computations, Pingouin (0.5.5) and Statsmodels (0.14.5) for conducting ANCOVA and Multivariate ANCOVA (MANCOVA), and Matplotlib (3.10.3) and Seaborn (0.13.2) for data visualization. ANCOVA was used to compare post-test scores between Experimental and Control groups, adjusting for pre-test covariates. Assumptions were checked for homogeneity of regression slopes, equality of residual variances (Levene’s test), and normality of residuals (Shapiro–Wilk test). MANCOVA with Pillai’s Trace assessed overall effects. Holm–Bonferroni correction was applied for multiple comparisons. Effect sizes are reported as partial eta squared ($\eta_{p}^{2}$), with significance level set at α=0.05.

The ANCOVA model was specified as:

$$Y_{i}=\beta_{0}+\beta_{1}\times {Group}_{i}+\beta_{2}\times X_{i}+\epsilon_{i}$$

where $Y_{i}$ represents the post-test score (dependent variable), ${\mathrm{Group}}_{i}$ is the categorical between-subject factor, and $X_{i}$ is the pre-test score (covariate).

#### 2.14.4. Assumption Checks

ANCOVA was employed to compare post-test scores between groups, adjusting for pre-test covariates. All key ANCOVA assumptions were examined and largely met: the interaction between group and baseline was non-significant, Shapiro–Wilk tests indicated normality of residuals, Levene’s tests mostly supported equality of variances, and Box’s M test for MANCOVA was non-significant.

## 3. Results

All analyses were conducted on a sample of 48 children (24 in the hippotherapy Experimental group and 24 in the waitlist Control group). Pre-test scores for each EF measure were included as covariates in the analyses of post-test outcomes. Exact *p*-values were adjusted using the Holm–Bonferroni correction to control for familywise error across the multiple EF outcomes.

### 3.1. Baseline Comparability

Given the non-random allocation of participants, baseline comparability between the Control and Experimental groups was assessed using standardized mean differences. Cohen’s d was calculated for continuous variables and Cramer’s *V* for the categorical variable (gender). As reported in Supplementary Table S4, the groups showed good baseline equivalence, with effect sizes consistently falling within the negligible-to-small range.

For demographic characteristics, Age yielded a small-to-medium effect size (*d* = 0.422), indicating that the Control group was slightly older on average, although this difference did not reach statistical significance (*p* > 0.05). While this represents a moderate difference, other demographic comparisons, including Gender distribution (Cramer’s *V* = 0.042), showed negligible association with group assignment. Across all pre-test EF measures, Cohen’s d values ranged from −0.205 (WCST Categories) to 0.195 (Stroop Congruent RT). The remaining measures—including WCST perseverative errors (*d* = 0.023), Corsi span (*d* = 0.057), Tower of London moves (*d* = 0.004), and Stroop Incongruent RT (*d* = −0.161)—also demonstrated negligible-to-small effect sizes. Importantly, none of the group differences reached statistical significance (all *p* > 0.05).

### 3.2. Executive Function (EF) Outcomes

Table 1 displays the raw pre-test and post-test means and standard deviations for each EF measure by group. By post-test, however, the Experimental group demonstrated generally better performance across the board. For example, the Experimental group completed more categories on the WCST (*M* = 4.70) than the Control group (*M* = 3.56), and made fewer perseverative errors (17.40 vs. 22.13). They also demonstrated greater working memory span (Corsi *M* = 4.52 vs. 4.26) and more efficient planning (Tower of London moves *M* = 41.88 vs. 46.14, where lower scores indicate better performance), as well as faster reaction times on the Stroop task (e.g., Incongruent RT *M* = 797.93 ms vs. 858.62 ms). These unadjusted differences suggest meaningful gains in EF for the hippotherapy group prior to adjusting for any baseline disparities (see Table 1).

#### 3.2.1. Multivariate Outcome

An initial multivariate analysis was conducted using a MANCOVA framework to examine the overall effect of the intervention on the battery of EF measures. Specifically, Pillai’s Trace was used as the test statistic. The results indicated a statistically significant overall effect of the intervention on the composite of EF measures when considering the MANCOVA output [Pillai’s Trace = 0.75, *F*(6,47) = 152.02, *p* < 0.001, partial *ηp*^2^ = 0.75]. This significant overall effect suggests that the intervention had a demonstrable impact across the EF domain. Consequently, to identify the specific EF measures influenced by the intervention, univariate ANCOVA analyses were performed, controlling for pre-intervention scores.

#### 3.2.2. Univariate Outcomes

To investigate the intervention’s impact on each specific EF component, ANCOVAs were performed, controlling for baseline scores. These analyses revealed statistically significant and robust differences between the intervention and Control groups across all measured EF domains after applying the Holm–Bonferroni correction for multiple comparisons (Table 2). Adjusted estimated marginal means (Table 3) consistently indicated higher post-test performance for the Experimental group after controlling for baseline differences. Intergroup adjusted mean differences ranged approximately from 0.4 to 1.0 units across EF tasks, confirming substantial intervention benefits. All effects were associated with large effect sizes (partial *ηp*^2^ = 0.47–0.86) and non-overlapping 95% confidence intervals between groups.

Specifically, significant effects were observed for cognitive flexibility, indexed by WCST categories completed (*F*(1,45) = 92.26, *p* < 0.001, partial *ηp*^2^ = 0.672), planning ability, as reflected in the Tower of London total moves (*F*(1,45) = 101.48, *p* < 0.001, partial *ηp*^2^ = 0.693), and working memory (Corsi span) (*F*(1,45) = 40.43, *p* < 0.001, partial *ηp*^2^ = 0.473).

The most pronounced improvements were observed in inhibitory control on the Stroop task, where the Experimental group demonstrated markedly faster responses than the Control group (congruent trials: *F*(1,45) = 254.12, partial *ηp*^2^ = 0.850; incongruent trials: *F*(1,45) = 265.80, partial *ηp*^2^ = 0.855; both *p*s < 0.001, Holm–Bonferroni-corrected).

Effect sizes were consistently large across all measures (all partial *ηp*^2^ ranging from 0.47 to 0.86), and observed power exceeded 0.99 for all significant findings. According to Cohen’s benchmarks (small = 0.01, medium = 0.06, large = 0.14), all observed partial *ηp*^2^ values (0.47–0.86) represent very large effects, highlighting the substantial magnitude of the intervention’s impact across EF domains. Furthermore, the 95% confidence intervals for the adjusted means did not overlap between groups, underscoring the reliability and practical significance of these intervention effects. Table 3 presents the corresponding adjusted post-test estimated marginal means and 95% confidence intervals. For instance, after baseline adjustment, the Experimental group completed on average one more category on the WCST than the Control group (adjusted *M* = 4.58, 95% CI [4.45, 4.72] vs. 3.67 [3.54, 3.81]). Likewise, on the Stroop Incongruent condition, the Experimental group’s adjusted mean reaction time was approximately 48 ms faster (804 ms [800, 809] vs. 852 ms [848, 856]).

Table 3 presents the adjusted post-test (estimated marginal) means for each group, controlling for baseline, along with their 95% confidence intervals. The adjusted means further illustrate the intervention’s benefits: for every EF outcome, the Experimental group’s estimated post-test performance was superior to that of the Control group. For instance, after adjusting for baseline differences, the Experimental group completed on average one more category on the WCST than the Control group (adjusted *M* = 4.58, 95% CI [4.45, 4.72] vs. 3.67 [3.54, 3.81] for controls). Similarly, on the Stroop Incongruent condition, the Experimental group’s adjusted mean reaction time was about 48 ms faster than the Control group’s (804 ms [800, 809] vs. 852 ms [848, 856]). In all cases, the 95% CIs for the two groups showed minimal to no overlap, underscoring the reliability and practical significance of these improvements.

Diagnostic tests supported the appropriateness of all ANCOVA models. Tests of homogeneity of regression slopes revealed no significant interactions between covariates and group (all *p* > 0.12), and Shapiro–Wilk tests indicated that residuals were normally distributed for all outcomes (all *p* > 0.21). These results confirm that model assumptions were satisfactorily met.

To further enhance the transparency of our findings and directly address potential concerns regarding model specification and data handling, we have included a series of supplementary visualizations in the appendix. Figure S1 provides a detailed view of individual participant trajectories and group-level changes from pre- to post-intervention for all six cognitive measures, illustrating the raw score distributions and patterns of change. Complementing these individual-level data, diagnostic analyses were performed to rigorously assess the assumptions underpinning our primary statistical models. Specifically, Figure S2 examines the linearity of the relationship between pre- and post-intervention scores for each outcome, while Figure S3 presents quantile–quantile (QQ) plots to evaluate the normality of the residuals from the ANCOVA models. These visualizations collectively support the appropriateness of the analytical approach and the reliability of the reported effect sizes.

### 3.3. EEG Outcomes

#### 3.3.1. Power Spectral Density (PSD)

The linear mixed-effects model revealed significant main effects of Group across most cortical regions and frequency bands (delta, theta, alpha, beta, and gamma; all $P_{\mathrm{FDR}}<0.05$). These findings indicate baseline differences in absolute power between the Experimental and Control groups, with the Experimental group generally showing higher power values (*Group* β coefficients were positive in all ROI × Band combinations). While these baseline differences were noted, our primary focus was on the intervention-induced changes, as detailed in the *Group* × *Time* interaction analyses below.

In contrast, main effects of Time were largely absent. After FDR correction, a significant Time effect emerged only in the frontal alpha-band ($\beta_{\mathrm{Time}}=0.165,\;P_{\mathrm{FDR}}=0.038$), suggesting a statistically reliable change in frontal alpha power over time, independent of group assignment. No other ROI × Band showed a significant Time effect at the FDR-corrected threshold.

Regarding Group × Time interactions, the analysis identified a single significant interaction in the frontal delta-band ($\beta_{\mathrm{Group}\times \mathrm{Time}}=-0.290,P_{\mathrm{FDR}}=0.032$). This negative interaction term indicates that delta power in the frontal region changed differentially over time between the Experimental and Control groups, consistent with a relative reduction (or smaller increase) in frontal delta power in the Experimental group following the intervention. No other cortical region or frequency band exhibited a significant Group × Time interaction after FDR correction.

**Figure 3:**
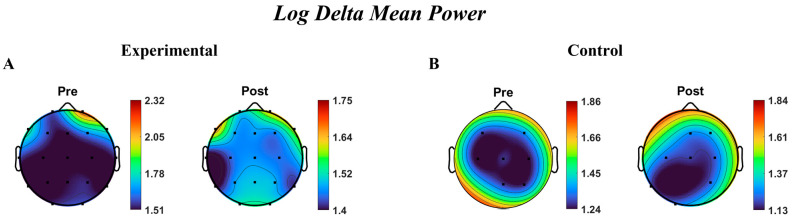
Scalp topographical maps of EEG delta-band (0.5–4 Hz) power in the pre- and post-conditions. (**A**) Experimental group: The left map represents the pre-intervention recording, and the right map represents the post-intervention recording. (**B**) Control group: The left map represents the pre-test recording, and the right map represents the post-test recording. The maps show the interpolated spatial distribution of mean delta power across scalp electrodes. Warmer colors represent higher delta power, while cooler colors indicate lower values.

Complete model estimates, standard errors, and test statistics are reported in Supplementary Table S6. Figure 3 displays scalp topographies of EEG delta power at pre- and post-intervention time points.

#### 3.3.2. Pairwise Spectral Correlations

The analysis of linear amplitude correlations revealed no significant main effects of Group after FDR correction across any frequency band or ROI pair (all $P_{FDR}>0.05$). In contrast, several main effects of Time survived FDR correction, particularly within the delta-band (e.g., prefrontal–parietal, prefrontal–temporal, frontal–parietal, frontal–occipital, central–parietal, central–occipital), indicating longitudinal increases in linear amplitude coupling across multiple large-scale cortical connections. Additional time-related effects were observed in the theta-band (e.g., prefrontal–parietal, prefrontal–temporal, frontal–occipital) and in several alpha-band connections.

Importantly, significant Group × Time interactions emerged in the alpha-band, specifically for the prefrontal–parietal ($\beta =-0.207,\;P_{FDR}=0.043$), prefrontal–temporal ($\beta =-0.208,\;P_{FDR}=0.043$), prefrontal–occipital ($\beta =-0.241,\;P_{FDR}=0.043$), and frontal–occipital ($\beta =-0.182,\;P_{FDR}=0.043$) connections. The negative interaction coefficients indicate a relative reduction in alpha-band linear coupling over time in the intervention group compared with the Control group, suggesting treatment-specific modulation of large-scale frontally mediated connectivity.

**Figure 4:**
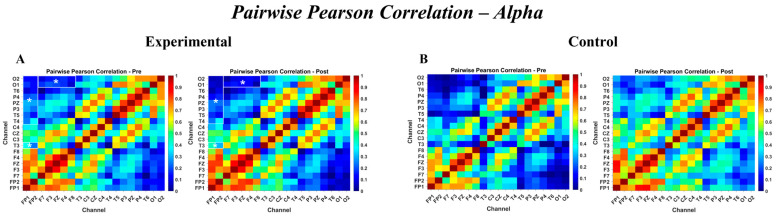
Pairwise Pearson correlation matrices between EEG channels in the alpha frequency band for the Experimental and Control groups. (**A**) Experimental group and (**B**) Control group. For each group, the left matrix represents the pre-intervention condition and the right matrix represents the post-intervention condition. Each matrix element represents the Pearson correlation coefficient between pairs of EEG channels. Warmer colors indicate stronger correlations, whereas cooler colors indicate weaker correlations according to the color scale. Connections that showed statistically significant Group × Time interactions are marked with an asterisk (*).

Complete model estimates, standard errors, and test statistics are reported in Supplementary Table S7. The correlation patterns are shown in Figure 4, which presents pairwise correlation matrices for the alpha frequency band.

#### 3.3.3. Phase Transfer Entropy (PTE) Connectivity

The results of PTE analysis showed no significant Group × Time interaction after FDR correction, indicating that the intervention did not induce differential changes in directed information flow relative to controls.

Nonetheless, significant main effects of Group were found primarily in the gamma frequency band, reflecting baseline differences in directed connectivity between groups. Meanwhile, significant main effects of Time were detected mostly in the alpha and beta bands, characterized by widespread reductions in directed information flow over the course of the study period.

Together, these findings suggest that although the intervention did not significantly modify PTE trajectories compared to controls, there were notable baseline group differences and a general decrease in information flow across multiple cortical connections over time.

Complete model estimates, standard errors, and test statistics are reported in Supplementary Table S8.

#### 3.3.4. Phase Lag Index (PLI) Connectivity

The linear mixed model (LMM) analysis of PLI values revealed significant findings for both interaction and main effects. Specifically, two significant Group × Time interactions were identified after FDR correction.

First, a significant interaction was observed in the frontal–temporal connection in the theta-band ($\beta =0.020,\;P_{FDR}=0.044$), indicating a significant increase in phase synchronization within the Experimental group over time relative to the Control group. For this connection, significant main effects were also detected, including a main effect of Group ($\beta =-0.022,\;P_{FDR}=0.0008$) and a main effect of Time ($\beta =-0.017,\;P_{FDR}=0.029$).

Second, a significant interaction was found in the prefrontal–occipital connection in the beta-band ($\beta =0.016,\;P_{FDR}=0.008$), indicating a differential change in phase coupling between groups over time. For this connection, neither the main effect of Group ($P_{FDR}=0.170$) nor the main effect of Time ($P_{FDR}=0.122$) reached statistical significance.

No other connections reached statistical significance for the interaction effect after FDR correction.

**Figure 5:**
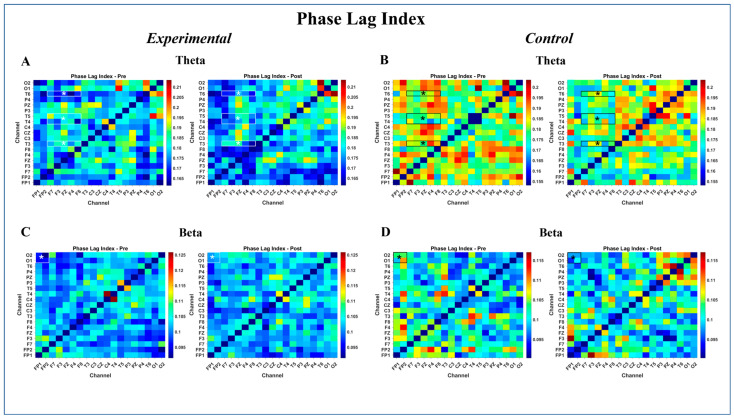
Pairwise Phase Lag Index (PLI) connectivity matrices between EEG channels for the Experimental and Control groups in the theta and beta frequency bands. (**A**,**C**) Experimental group and (**B**,**D**) Control group. Panels (**A**,**B**) show the theta-band, while panels (**C**,**D**) show the beta-band. For each panel, the left matrix represents the pre-intervention condition and the right matrix represents the post-intervention condition. Each matrix element represents the PLI between a pair of EEG channels. Warmer colors indicate stronger functional connectivity, whereas cooler colors indicate weaker connectivity. Connections that showed statistically significant changes are marked with an asterisk (*).

The PLI connectivitypatterns are shown in Figure 5, which presents PLI matrices for the theta and beta frequency bands.

Complete model estimates, standard errors, and test statistics are reported in Supplementary Table S9.

**Figure 6:**
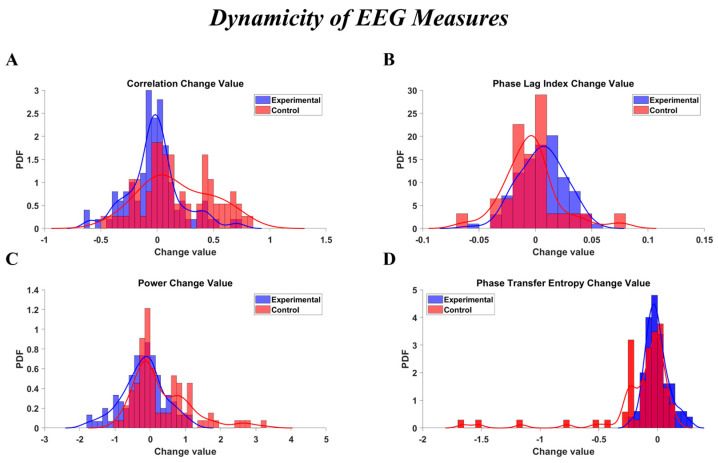
Dynamicity of EEG connectivity and power measures before and after hippotherapy in children with ASD. (**A**) Probability density function of change values (post − pre) for correlation in the Experimental and Control groups; (**B**) probability density function of change values (post − pre) for PLI; (**C**) probability density function of change values (post − pre) for power; (**D**) probability density function of change values (post − pre) for PTE. Note: Curves represent the distribution of post–pre change values for each measure in both groups.

Figure 6 provides a visual summary of changes in EEG connectivity and power measures following the 12-session hippotherapy program. The distributions of post–pre change values are shown for correlation, PLI, PTE, and power in both the Experimental and Control groups. Overall, the figure highlights group-specific differences in the dynamicity of EEG measures after the intervention.

## 4. Discussion

This study investigated the effects of a 12-session hippotherapy program on EFs and EEG in children with ASD, following the protocol detailed in the Methods Section. The intervention was conducted in a controlled arena environment, utilizing horses selected for their calm temperament and suitability for therapeutic interactions. Children in the hippotherapy group displayed narrower distributions of pre–post EEG changes, primarily in frontal and prefrontal regions, compared with the Control group. This reduced spread of change values (Figure 3, Figure 4, Figure 5 and Figure 6) suggests relatively more uniform modulation patterns over time, rather than an overall enhancement or suppression of activity. Given the presence of baseline EEG differences noted in certain frontal areas, these consistency patterns should be interpreted as relative rather than absolute stabilization. The relative consistency of EEG signals in the therapy group is compatible with the idea that structured sensory–motor engagement during hippotherapy could promote more consistent patterns of neural coordination. However, this interpretation should be viewed as tentative, given the limited sample size and absence of task-based neural data. The specific characteristics of the equine partners, selected for their calm temperament (temperament score of 1–2), consistent gait, appropriate stature (ranging from 14.0 to 16.1 hands), and prior extensive therapeutic experience (ranging from 2 to 8 years), alongside the structured and contained nature of the arena space, likely contributed to creating an optimal therapeutic environment where these neural and behavioral changes were observed.

Behaviorally, children who received hippotherapy demonstrated robust improvements across core executive domains (cognitive flexibility, response inhibition, working memory, and planning) compared with controls. These gains remained after adjusting for baseline scores and were associated with large effect sizes. This trial yielded notably large effect sizes for several EF outcomes (partial η^2^ ranging from 0.47 to 0.86). While these findings suggest a significant potential impact of hippotherapy, definitive causal claims require careful consideration due to the study’s design. Specifically, the non-randomized group assignment, influenced by parental preference and scheduling, introduces potential selection bias. Coupled with a waitlist control, this means that observed improvements may reflect non-specific factors beyond the core therapeutic mechanisms. These factors include participant expectations, the intervention’s inherent appeal and novelty, and enhanced engagement from a motivated cohort (evidenced by perfect attendance and no dropouts). Repeated assessments may also contribute to learning or adaptation effects, potentially amplified in the intervention group due to its greater appeal. Therefore, while promising, these results should be interpreted cautiously, acknowledging the contribution of these non-specific elements. Our behavioral results align with prior randomized controlled trials and smaller experimental studies that report benefits of equine-assisted interventions for children with ASD [12] and with smaller, well-controlled studies that reported EF or adaptive gains following structured equine programs [47]. Moreover, similar EF benefits have been observed in non-ASD samples following equine-assisted activities [14], which supports the broader plausibility that equine interventions can target cognitive systems relevant to EF; however, those non-ASD findings should not be assumed to generalize directly to ASD without caution. Collectively, these robust behavioral improvements suggest a promising role for structured hippotherapy, as implemented in our protocol, as a potential intervention strategy for enhancing core EFs in children with ASD, thereby contributing valuable evidence to the field of hippotherapy.

At the same time, systematic reviews and meta-analyses in this area emphasize considerable heterogeneity in study protocols, outcome measures, and sample characteristics, which limits strong generalization from any single trial [16,17]. Accordingly, while our behavioral data add to a converging set of findings that suggest EAT can improve social, adaptive, and executive outcomes in ASD, readers should interpret effect magnitudes in the context of a literature that still varies widely in quality and consistency.

Closer inspection of the linear mixed model results indicates that the observed group differences were not uniformly distributed across the brain but were concentrated within specific networks and frequency bands. Linear mixed model analyses revealed spatially selective Group × Time interactions—particularly decreases in frontal delta power, reductions in alpha-band correlation among frontally anchored connections, and increases in theta and beta phase-locking (PLI) between frontal–temporal and prefrontal–occipital regions. These distributed yet frontal-centered effects represent differential change patterns over time between groups, after accounting for baseline offsets within the model. The spatial concentration of these interaction effects suggests that the neural modulation observed following hippotherapy may preferentially involve circuits supporting executive control, sensory integration, and large-scale coordination.

Importantly, the spatial pattern of these effects overlaps with cortical systems commonly associated with executive and regulatory processes. Frontal and prefrontal regions play a central role in cognitive control, response inhibition, and working memory [2,48], while fronto-temporal interactions contribute to the integration of sensory and higher-order cognitive information within distributed cortical networks [49,50]. The presence of significant interaction effects within these networks suggests that the intervention may have influenced the temporal organization of activity in circuits that are functionally relevant for executive processing, although the present resting-state data cannot directly establish task-specific mechanisms.

Behavioral assessments in the present study demonstrated significant improvements following the hippotherapy intervention, indicating that the program produced measurable functional benefits in children with ASD. In addition to these behavioral outcomes, EEG analyses were conducted to examine whether the intervention was also associated with measurable changes in neural activity and connectivity patterns.

The results showed significant changes in several EEG measures after the intervention, suggesting that hippotherapy may be accompanied by detectable modifications in resting-state brain dynamics. In addition to these focal interaction effects, the hippotherapy group exhibited a narrower range of pre–post changes across sessions, which can be interpreted as a pattern of reduced variability rather than monotonic normalization. This pattern fits within theoretical frameworks suggesting that short-term consistency in resting-state spectral profiles may reflect the maintenance of stable excitability and coordination levels [18]. However, given the presence of pre-existing between-group differences in baseline spectral profiles, these patterns likely reflect relative adjustments rather than full normalization. Within this context, greater within-subject consistency across sessions may reflect a more regulated or organized baseline neural state following the intervention.

At the same time, the literature on neural variability in ASD remains complex, with studies reporting both increased and decreased variability depending on the experimental conditions and analytic approaches used [21]. Therefore, the stability patterns observed here should be interpreted cautiously and considered preliminary. Nevertheless, these findings suggest that resting-state EEG measures may provide useful complementary information about neural processes associated with hippotherapy interventions in ASD. Further studies with larger samples and longitudinal designs will be necessary to better understand the neural mechanisms underlying these effects and to determine whether such EEG markers could serve as reliable indicators of intervention-related neural change.

Several methodological points are relevant to interpreting the neural data. Resting-state EEG offers a convenient window into ongoing brain dynamics, but it is a coarse measure: oscillatory power during rest more often indexes arousal, vigilance, or system efficiency than the moment-to-moment processes required by executive tasks. Levin et al. (2020) show that brief resting recordings can yield reliable test–re-test spectral signatures, but they also highlight the need to relate those signatures to behavior before treating them as unambiguous markers of “normalization” [18]. Likewise, Hecker et al. (2022) underscore that EEG variability in ASD depends on temporal scale, task context, and analytic choice [21]. In short, resting-EEG stability in our trial is an informative finding, but task-evoked EEG (event-related potentials and time–frequency responses during Go/No-Go, Stop-Signal, Flanker, or N-back tasks) and multimodal imaging would be essential for establishing a direct link to the EF improvements we observed. This underscores a critical need for future research in hippotherapy to employ multimodal neurophysiological assessments that integrate resting-state data with task-based measures and potentially other neuroimaging techniques to fully elucidate the neural mechanisms underlying the observed behavioral improvements.

Importantly, the robust behavioral improvements seen here, when considered alongside the mixed neural findings, suggest that the precise mechanisms driving EF gains require further elucidation. The observed resting-state EEG stability co-occurred with behavioral improvements, but the direct causal pathway remains to be fully specified. It is possible that hippotherapy induced subtle or spatially focal neural changes that were not captured by our resting-state metrics, or that individual differences in neurophysiology, age, or baseline EF moderated neural responsiveness to the intervention. Moreover, as systematic reviews note, the EAAT literature has many small studies with variable methods, and pooled estimates can be influenced by publication bias and heterogeneity [16,17]. Our results echo these broader concerns: while encouraging, they underline the need for multimodal, standardized protocols to elucidate mechanisms.

Our results from the linear mixed model showed that the EEG changes in the hippotherapy group had narrower and more centralized distributions. In contrast, the Control group displayed broader and more variable changes (Figure 5). This supports earlier findings that children with ASD show unusually high variability in connectivity over time and space. For example, Kang et al. (2023) reported that EEG connectivity diversity is higher in ASD compared to neurotypical controls [43]. Autism is often marked by unstable and noisy brain dynamics, which can hinder efficient information processing [51]. In this context, hippotherapy was associated with a tendency toward less dispersed change distributions, which corresponds with a more consistent and regulated neural state.

The differences between the groups were particularly involving frontal and fronto-temporal networks and, in some cases, prefrontal–occipital interactions (Figure 3, Figure 4 and Figure 5). The frontal and prefrontal cortices play a key role in executive control and attention regulation, while the occipital regions are important for visual and sensory processing. These are areas often disrupted in ASD [37]. The observed reduction in change variability within frontal and fronto-temporal networks may reflect a trend toward more constrained neural fluctuation patterns, potentially indicating a partial modulation of circuits that often show excessive dynamical range in ASD. While this does not confirm “stabilization” per se, it suggests a shift toward more consistent signal modulation within executive and sensory hubs. These findings are particularly significant for the hippotherapy field as they provide potential neural correlates for the observed behavioral gains, suggesting an association between hippotherapy and modulation of neural circuits critical for EF and sensory processing in ASD. Such evidence is vital for suggesting the efficacy of these therapies and informing the direction for future intervention development.

One interesting idea is that excessive neural variability in frontal networks might create a functional bottleneck in ASD. This can overload executive systems and make flexible communication difficult. This might explain why some autistic individuals struggle with social communication and task-switching but do well in areas like visuospatial reasoning or math processing. These skills may benefit from specialized local networks and less global integration [52]. By being linked to lower temporal variability and more coordinated sensory–motor engagement, hippotherapy may contribute to rebalancing the interplay between local and global neural dynamics—a possibility that warrants direct testing in future multimodal experiments.

Overall, the reduced variability in important executive and sensory areas is associated with efficient and stable brain function in children with ASD. These findings support behavioral studies showing that horse-based therapies improve communication and self-regulation in ASD [53]. The rhythmic and multisensory input from riding may be linked to the alignment of cortical rhythms, improvement in large-scale coordination, and normalization of unusual excitability patterns [54,55]. Collectively, our findings suggest that hippotherapy is a promising avenue that warrants further investigation for its potential in improving both neural regulation and EFs in children with ASD. Taken together, the EEG findings indicate measurable modulation of resting-state dynamics associated with hippotherapy, characterized by reduced variability of change distributions and localized interaction effects within prefrontal-centered networks. Although preliminary, these neural patterns complement the robust behavioral improvements observed, offering an initial mechanistic insight into how structured equine engagement might influence executive circuits in ASD. Future research with larger, multimodal designs will be crucial to confirm these effects and to disentangle the precise contributions of sensory, motor, and attentional components.

### 4.1. Limitations

This study is subject to several limitations that warrant careful consideration when interpreting the findings.

Firstly, the demographic profile of our sample inherently constrains the generalizability of our conclusions. Participants were exclusively children aged 9–12 years with an IQ exceeding 70. While this homogeneity facilitated meaningful engagement with the assessments and intervention, it means our findings may not directly apply to younger children, older age groups, or individuals with lower cognitive functioning, including those with co-occurring intellectual disabilities. Future research is needed to explore the efficacy of hippotherapy across these broader or distinct populations.

Secondly, regarding the Control group’s protocol, while we implemented a time- and attention-matched approach to mitigate non-specific factors such as therapist interaction and time out of routine, the control intervention was not an active alternative precisely matched for novelty or specific physical activity levels. This design choice means that while we aimed to control for general therapeutic attention, potential confounding influences related to the unique aspects of the control activities cannot be entirely ruled out.

Thirdly, the limited number and specific characteristics of the horses, while ensuring consistency and reducing variability within the intervention, also constrain the generalizability of our findings. Working with five horses of comparable health, temperament, and therapeutic experience helped create a controlled study environment; however, this small and homogeneous sample restricts our ability to examine the influence of individual equine traits on therapeutic outcomes. Although preliminary observations suggested possible links between certain behavioral or physical characteristics and patient progress, the absence of standardized quantitative measures of equine temperament, combined with the limited sample size, prevented formal statistical analysis. Future studies incorporating larger and more diverse equine populations, randomized horse–participant assignments, and validated measures of equine attributes will be essential for clarifying the nuanced contributions of horse-related factors to therapeutic effectiveness.

Fourthly, our EEG analyses were limited to resting-state recordings. Without employing task-evoked EEG or other neuroimaging techniques, we are unable to precisely localize the neural circuitry that underlies the observed behavioral changes.

Finally, while the non-randomized nature of our study design limits definitive causal inference, the promising neural and behavioral findings warrant further investigation into the underlying mechanisms. Specifically, understanding how executive control networks respond to hippotherapy requires addressing the challenge that observed changes may stem from both specific therapeutic effects and non-specific factors like novelty and therapeutic engagement. Although both groups underwent pre- and post-intervention assessments, the non-randomized allocation introduces potential selection bias. Furthermore, the repeated nature of assessments might contribute to learning or adaptation effects. Critically, differences in participant motivation and the inherent novelty and engagement of the hippotherapy intervention itself, compared to the control activities, could have amplified the observed improvements in the intervention group. Therefore, a definitive disentanglement of these specific therapeutic effects from non-specific factors necessitates future research employing randomized allocation, blinded assessments, and larger, diverse samples to establish clearer causal relationships.

### 4.2. Future Directions

Future research should prioritize well-powered randomized controlled trials with active control conditions and blinded assessments. Integrating multimodal neural measures, such as resting-state and task-evoked EEG along with fMRI, is crucial to determine how hippotherapy specifically impacts neural dynamics supporting executive control and behavior in ASD. To clarify mechanisms and strengthen causal inference, future research should prioritize larger, well-powered randomized controlled trials with active control conditions (e.g., comparable physical activity without horses), standardized hippotherapy protocols, and blinded outcome assessment. Critically, these trials should include multimodal neural measures, for example, combining longitudinal resting-EEG with task-evoked EEG, fMRI, and physiological stress markers, to determine whether resting-state stability maps onto task-related neural changes that support executive control. Given the observed resting-state EEG stability and its correlation with behavioral improvements in our study, these integrated designs are strongly suggested as essential for establishing how equine-assisted approaches affect brain and behavior in ASD.

Furthermore, to deepen our understanding of the therapeutic process, future investigations should explicitly examine the nuanced role of equine partners. This exploration could involve designing studies that leverage larger and more diverse equine populations, enabling statistical analyses to elucidate how specific factors (such as age, breed, and importantly, quantitatively assessed temperament) may influence patient progress. For instance, future research should aim to develop and validate objective measures of equine temperament (e.g., using behavioral checklists or validated scales), allowing for a statistical exploration of observations such as those qualitatively noted in our study, where calmer horses appeared to correlate with enhanced patient outcomes. Moreover, incorporating randomized assignment of participants to specific horses would be invaluable for controlling potential biases and isolating the effects attributable to individual equine partners. Crucially, the development and validation of objective measures for equine engagement and welfare within the therapeutic context are needed to ensure their active and willing participation, which could further enhance therapeutic effectiveness. Finally, exploring potential interactions between patient characteristics and equine attributes holds promise for personalizing hippotherapy interventions and maximizing therapeutic benefits.

## 5. Conclusions

In sum, a 12-session hippotherapy intervention was associated with significant improvements in EFs and resting-EEG stability in children with ASD. While these findings suggest a potential role for hippotherapy in engaging regulatory brain mechanisms that may be associated with cognitive enhancement, due to the quasi-experimental design, we caution against definitive causal claims, emphasizing that observed associations, particularly neural stabilization in frontal regions, require further investigation. The careful selection of our equine partners, while contributing to the consistency of the intervention, also highlights the need for future research to explore how specific equine characteristics might influence these positive outcomes. Future rigorous research, specifically randomized controlled trials with standardized protocols and multimodal assessments, is crucial to confirm these effects, elucidate underlying neural pathways, and solidify the evidence base for hippotherapy by better understanding the critical role of the equine partner in clinical practice.

## Figures and Tables

**Table 1:** Raw means (M) and standard deviations (SDs) by Group and Time (*N* = 24 per group).

Measure	Pre-Test Control (*M* ± SD)	Pre-Test Experimental (*M* ± SD)	Post-Test Control (*M* ± SD)	Post-Test Experimental (*M* ± SD)
WCST Categories (no.)	3.46 ± 0.93	3.71 ± 1.08	3.56 ± 0.95	4.70 ± 1.03
WCST Perseverative Errors (no.)	22.31 ± 3.84	22.03 ± 5.11	22.13 ± 4.41	17.40 ± 5.51
Corsi Span (blocks)	4.08 ± 0.65	3.88 ± 0.61	4.26 ± 0.68	4.52 ± 0.62
Tower of London—Total Moves	45.95 ± 4.71	46.71 ± 4.16	46.14 ± 5.08	41.88 ± 4.28
Stroop Congruent RT (ms)	569.10 ± 52.65	565.74 ± 49.34	569.48 ± 53.88	532.10 ± 49.59
Stroop Incongruent RT (ms)	860.19 ± 61.45	847.21 ± 92.08	858.62 ± 61.55	797.93 ± 93.81

Note: *M* = mean; SD = standard deviation. *N* = 24 per group. For all measures, lower scores indicate better performance on efficiency-based tasks (e.g., Tower of London moves, Stroop reaction times); higher scores indicate better performance on capacity-based tasks (e.g., WCST categories, Corsi span).

**Table 2:** ANCOVA results for Group (adjusted for pre-test covariates).

Measure	F(1,45)	*p* (Exact)	*p* (Holm)	Partial η^2^	Observed Power
WCST Categories	92.26	0.000	0.000	0.672	1.000
WCST Perseverative Errors	101.76	0.000	0.000	0.693	1.000
Corsi Span	40.43	0.000	0.000	0.473	1.000
Tower of London Moves	101.48	0.000	0.000	0.693	1.000
Stroop Congruent RT	254.12	0.000	0.000	0.850	1.000
Stroop Incongruent RT	265.80	0.000	0.000	0.855	1.000

Note: ANCOVAs include each measure’s pre-test score as a covariate. *p*-values are Holm–Bonferroni-adjusted for multiple comparisons. Partial η^2^ = partial eta squared effect size. Observed power computed at α = 0.05.

**Table 3:** Adjusted post-test means (estimated marginal means) and 95% confidence intervals.

Measure	Control Adjusted Mean (95% CI)	Experimental Adjusted Mean (95% CI)
WCST Categories	3.67 (3.54, 3.81)	4.58 (4.45, 4.72)
WCST Perseverative Errors	21.98 (21.35, 22.60)	17.55 (16.93, 18.18)
Corsi Span	4.16 (4.06, 4.26)	4.62 (4.52, 4.72)
Tower of London Moves	46.51 (45.81, 47.22)	41.51 (40.80, 42.22)
Stroop Congruent RT	567.79 (564.76, 570.83)	533.79 (530.75, 536.83)
Stroop Incongruent RT	852.09 (847.94, 856.25)	804.46 (800.31, 808.61)

Note: Adjusted means are estimated marginal means from ANCOVA, evaluated at the grand mean of each baseline covariate. For all measures, lower scores indicate better performance on efficiency-based tasks (e.g., Tower of London moves, Stroop reaction times); higher scores indicate better performance on capacity-based tasks (e.g., WCST categories, Corsi span).

## Data Availability

The data supporting the findings of this study are not publicly available due to ethical and privacy restrictions related to the protection of participant confidentiality and will not be deposited in a public repository. During the peer-review process, the datasets are available to editors and reviewers upon request. Following acceptance and publication, the data will be made available to qualified researchers for academic research purposes upon reasonable request from the corresponding author, subject to compliance with institutional ethical guidelines and, where applicable, the completion of a data-use agreement.

## Supplementary Materials

The following supporting information can be downloaded at: https://www.mdpi.com/article/10.3390/brainsci16040413/s1. Table S1 provides an overview of the 12-session hippotherapy protocol. Table S2 details the characteristics of the therapy horses (*n* = 5), and Table S3 outlines staff training and credentials. Safety and equine welfare are addressed in File S1 (pre/during/post-session checklist), and session fidelity is monitored using the checklist in File S2. Further supporting data include Table S4—Standardized mean differences (Cohen’s d) for baseline variables; Table S5—Participant accommodations during cognitive testing; Table S6—Linear mixed-effects model results for Power Spectral Density; Table S7—Linear mixed-effects model results for pairwise Pearson correlation coefficients; Table S8—Linear mixed-effects model results for Phase Transfer Entropy; Table S9—Linear mixed-effects model results for Phase Lag Index. Visualizations are also provided: Figure S1 displays group differences in cognitive performance pre- and post-intervention; Figure S2 shows linearity assessment of pre- to post-intervention changes; and Figure S3 illustrates normality assessment of model residuals via QQ plots.

## Abbreviations

The following abbreviations are used in this manuscript:

Abbreviation	Term
AHA	American Hippotherapy Association
ASR	Artifact Subspace Reconstruction
ASD	Autism Spectrum Disorder
EEG	Electroencephalography
EAT	Equine-Assisted Therapy
EF	Executive Function
FDR	False Discovery Rate
ICA	Independent Component Analysis
nPTE	Normalized Phase Transfer Entropy
PLI	Phase Lag Index
PTE	Phase Transfer Entropy
PSD	Power Spectral Density
PDF	Probability Density Function
RTs	Reaction Times
ROI	Region Of Interest
TE	Transfer Entropy
WCST	Wisconsin Card Sorting Test
