# Supplementary material for: Hippotherapy for Children with Autism Spectrum Disorder: Executive Function and Electrophysiological Outcomes
Source: Brain Sci. 2026 Apr 14;16(4):413. doi: 10.3390/brainsci16040413 (PMC13114843; doi:10.3390/brainsci16040413)
Supplement: Supplementary file 1 [file brainsci-16-00413-s001.zip › brainsci-4194834-supplementary.pdf]

Supplementary Material

The following supplementary tables and figures accompany the main manuscript:

Tables:

- Table S1. Overview of the 12-Session Hippotherapy Protocol.
- Table S2. Therapy horse characteristics (n = 5)
- Table S3. staff training & credentials.
- Table S4. Standardized Mean Differences (Cohen's d) for Baseline Variables.
- Table S5. Participant Accommodations During Cognitive Testing.
- Table S6. Linear mixed-effects model results for power spectral density.
- Table S7. Linear mixed-effects model results for pairwise Pearson correlation coefficients.
- Table S8. Linear mixed-effects model results for phase transfer entropy.
- Table S9. Linear mixed-effects model results for phase lag index.

Files:

- File S1. Safety & Equine Welfare checklist (pre/during/post-session)
- File S2. Fidelity checklist (use one per session).

Figures:

- Figure S1. Group Differences in Cognitive Performance Pre- and Post-Intervention
- Figure S2. Linearity Assessment of Pre- to Post-Intervention Changes
- Figure S3. Normality Assessment of Model Residuals via QQ Plots

Tables:

Table S1. Overview of the 12-Session Hippotherapy Protocol

Session	Goal	Main Activity	Core Task/Description	Targeted Skills
**1**	Orientation and relationship building	Introduction to horse and stable environment	Meet the horse, learn safety and hygiene rules, and explore the stable	Emotional connection, trust, communication
**2**	Developing responsibility and sensory connection	Grooming the horse	Identify tools, practice brushing and care routines	Sensory integration, fine motor control, empathy
**3**	Nurturing and feeding	Feeding activities	Prepare and feed safe horse snacks ("horse pizza")	Responsibility, sequencing, social interaction
**4**	Creative expression and care	Painting and washing	Decorate with safe paints, then wash and care for horse	Creativity, cooperation, motor planning
**5**	Coordination (off-horse)	Ball and hoop games	Throw/catch at varied distances and targets	Gross motor control, planning, attention
**6**	Mounted introduction	First horseback experience	Learn mounting/dismounting, balance in static position	Balance, body awareness, confidence
**7**	Flexibility and balance	Stretching on horseback	Guided stretching while mounted (static and walking)	Postural control, flexibility
**8**	Coordination (on-horse I)	Ball and hoop tossing (large targets)	Toss objects into large targets while horse walks	Bilateral coordination, focus
**9**	Coordination (on-horse II)	Ball and hoop tossing (medium targets)	Progressively smaller targets and increased distance	Eye–hand coordination, spatial awareness
**10**	Coordination (on-horse III)	Ball and hoop tossing (small targets)	Randomized target sequence	Motor precision, adaptability
**11**	Autonomy and leadership	Leading the horse	Practice guiding horse with verbal and physical cues	Initiative, communication, self-control
**12**	Integration and closure	Navigation and farewell	Lead horse through path, group reflection and photos	Planning, social bonding, emotional closure

Note. *The intervention comprised two integrated phases: a foundational off-horse phase (Sessions 1–5) focusing on building rapport, sensory preparation, and responsibility, and a mounted hippotherapy phase (Sessions 6–12) utilizing the horse's movement for direct sensorimotor and therapeutic benefit.*

Table S2. Therapy horse characteristics (n = 5)

Horse ID	Breed	Age (years)	Sex	Height (hands)	Temperament score (1 = very calm, 5 = reactive)	Prior therapeutic experience (years)	Primary therapeutic use / notes
H1	Haflinger Horse	12	Gelding	16.0	1	6	Regular hippotherapy; calm with side-walkers
H2	Hanoverian Horse	9	Mare	15.2	2	4	Used for balance and core work; responsive to verbal cues
H3	Haflinger	14	Gelding	16.1	1	8	Longstanding therapy horse, low reactivity
H4	Connemara pony	11	Gelding	14.0	2	3	Suitable for smaller participants; steady walk rhythm
H5	Thoroughbred cross	10	Mare	15.3	2	2	Newer to program; monitored closely, substituted twice (dates logged)

Notes: *Temperament score: assigned by the equine manager and treating therapist at baseline using a standardized observational checklist (1 = very calm/steady; 5 = highly reactive/unpredictable). - Soundness & health: All horses received routine veterinary care and farrier management prior to and during the study period; no lameness or medical contraindications were observed during the trial. - Substitutions: When a primary horse was unavailable (illness, veterinary appointment, or scheduling conflict), a documented substitution occurred; each substitution was recorded with date and brief reason in the study log.*

Table S3. staff training & credentials

StaffID	Role	Primary Qualification / Profession	Relevant Training / Courses Completed	Certification Level (e.g., AHA)	License # (if applicable)	Years of EAI Experience	Date of Last Training / Refresher
	(e.g., Lead Therapist, Handler)	(e.g., OT, Psychologist, Equine Specialist)	(e.g., AHA Safety, First Aid, Equine Handling, Study-Specific Protocol Training)	(e.g., Basic, Advanced)	(e.g., State License #)		

This table details the qualifications, training, and experience of the staff involved in the study's equine-assisted interventions (EAI). All personnel listed have undergone the necessary training and hold relevant certifications to ensure participant safety and adherence to study protocols.

- StaffID: Unique identifier for each staff member.
- Role: Specific role of the staff member within the EAI sessions (e.g., Lead Therapist, Handler, Side-walker, Independent Observer).
- Primary Qualification / Profession: The staff member's main professional qualification or degree (e.g., Occupational Therapist, Psychologist, Equine Specialist).
- Relevant Training / Courses Completed: A list of specific training and courses undertaken, relevant to EAI, equine handling, and participant safety (e.g., AHA best-practice and safety course, First aid for equine settings, specific study protocol training).
- Certification Level (e.g., AHA): The level of any formal certifications obtained (e.g., Basic, Advanced AHA certification level).
- License # (if applicable): The professional license number, if applicable (e.g., State license number for therapists).
- Years of EAI Experience: Total number of years of professional experience in equine-assisted interventions.
- Date of Last Training / Refresher: The date of the most recent relevant training or refresher course completed.

Sample: Staff training & credentials log (sample summary — de-identified)

StaffID	Role	Primary Qualification / Profession	Relevant Training / Courses Completed	Certification Level (e.g., AHA)	License # (if applicable)	Years of EAI Experience	Date of Last Training / Refresher

**T01**	Lead Therapist	PhD Motor Behavior; Licensed OT/Physio	Local hippotherapy CE; CPR/First-aid; Child safeguarding	Equivalent AHA I–II	Licensed OT/Physio	12 years	2024-09 (annual refresh)
**A01**	Assistant / Side-walker	Diploma Equine Handling	AHA safety module (local)	Local	N/A	3 years	2024-06
**A02**	Assistant / Side-walker	Certified Riding Instructor (Level Basic)	First-aid for Children	N/A	N/A	4 years	2024-07
**A03**	Lead Handler	Professional Groom / Handler	Equine Welfare & Handling Course	N/A	N/A	8 years	2024-08
**O01**	Independent Observer	MSc Rehabilitation	Monitor Training (Fidelity Checklist Use)	N/A	N/A	2 years	2024-09

Staff Training and Credentials Summary:

- **AHA Best-Practice & Safety Overview:** Completed by all staff (or local equivalent).
- **First Aid & CPR for Children:** Completed by T01, A02, A03.
- **Equine Handling & Welfare Basics:** Completed by A01, A03.
- **Annual Refresher & Crisis-Response Drill:** Completed by all staff in 2024.

Table S4. Baseline Comparability: Demographic and Pre-test Executive Function Measures

Measure	Group	N	Mean (SD) or %	Cohen's d / Cramer's V	p-value
**Demographics**					
**Age**	Control	24	10.67 (1.17)	d = 0.422	0.145
	Experimental	24	10.17 (1.20)		
**Gender**	Control	24	58.33% Female	V = 0.042	0.772
	Experimental	24	50.00% Female		

**Executive Functions (Pre)**					
**WCST Categories**	Control	24	3.42 (1.02)	d = -0.205	0.481
	Experimental	24	3.62 (1.01)		
**WCST Perseverative Errors**	Control	24	22.23 (3.59)	d = 0.023	0.936
	Experimental	24	22.13 (5.06)		
**Corsi Span**	Control	24	3.96 (0.69)	d = 0.057	0.845
	Experimental	24	3.92 (0.78)		
**Tower of London Moves**	Control	24	45.90 (4.67)	d = 0.004	0.990
	Experimental	24	45.88 (5.01)		
**Stroop Congruent RT**	Control	24	567.56 (59.60)	d = 0.195	0.502
	Experimental	24	556.31 (55.52)		
**Stroop Incongruent RT**	Control	24	850.71 (68.23)	d = -0.161	0.579
	Experimental	24	864.09 (95.22)		

Notes: *Data are presented as Mean (Standard Deviation) or percentage. p-values indicate the statistical significance of differences between groups. Cohen's d and Cramer's V are effect size measures for mean differences and categorical differences, respectively.*

Table S5. Participant Accommodations During Cognitive Testing

Participant ID	Cognitive Test	Accommodation Applied	Reason for Accommodation	Examiner Notes
**S01**	WCST	Instructions repeated; Extended time for task initiation	To ensure full comprehension of multi-step instructions; To allow for more deliberate processing.	Participant was right-handed. Screen brightness was set to a moderate level. Standard seating distance was maintained. Initially required some prompting to begin, but demonstrated good engagement throughout.
**S01**	Corsi Block-Tapping Test	Brief task demonstration; Instructions repeated	To clarify the sequence-matching procedure; To confirm understanding of the task requirements.	Laptop mouse access was provided for response input. Chair height was adjusted to suit the child's posture.
**S01**	Stroop Color–Word Test	Visual aid for color-word mapping; Instructions repeated	To aid in understanding the task's core conflict (color	The participant appeared slightly fatigued but maintained focus to complete the task.

			vs. word); To ensure procedural clarity.	
**S01**	Tower of London Test	Brief task demonstration; Extended time for task initiation	To clarify the rules for moving discs between pegs; To ensure procedural understanding.	Initial execution speed was slower, but improved with minimal guidance, showing good problem-solving progression.
**S07**	WCST	Extended time for task initiation; Screen contrast adjusted	To accommodate a potentially slower information processing pace; For visual comfort.	Child was left-handed. Additional time was provided for each card to ensure comprehension.
**S07**	Corsi Block-Tapping Test	Brief task demonstration; Instructions repeated	To clarify the sequence-matching procedure; To reduce potential test anxiety.	Required a few moments of calming support during the assessment.
**S07**	Stroop Color–Word Test	Visual aid for color-word mapping; Laptop mouse access	To facilitate understanding of the color-word association; For ease of interaction with the test interface.	Monitor contrast was adjusted for optimal viewing. Participant responded relatively quickly.
**S07**	Tower of London Test	Brief task demonstration; Appropriate seating	To clarify movement rules; To ensure physical comfort during the task.	Appeared engaged and interested in the task.
**S12**	WCST	Extended time for task initiation; Instructions repeated; Mother seated nearby to support and maintain focus	To allow for more deliberate processing; To ensure full procedural understanding; To reduce distress and prevent leaving the seat.	Right-handed. Screen brightness was set to normal. Occasional repetition of instructions was needed.
**S12**	Corsi Block-Tapping Test	Brief task demonstration; Instructions repeated; Mother seated nearby to support and maintain focus	To clarify the sequence-matching procedure; To ensure procedural understanding; To reduce distress and prevent leaving the seat.	Seating and distance from the monitor were appropriate. Demonstrated good participation.
**…**	…	…	…	…

Table S6. Linear mixed-effects model results for power spectral density. Fixed effects of Group, Time, and Group × Time are reported for each ROI and frequency band. The table presents regression coefficients (β), standard errors (SE), and false discovery rate–corrected p-values (p_{DR}) (n = 48).

ROI	Band	Group p_{FDR}	Group β	Group SE	Time p_{FDR}	Time β	Time SE	Group:Time p_{FDR}	Group:Time β	Group:Time SE
**Prefrontal**	Delta	0.001316	0.540547	0.161266	0.521238	-0.10028	0.11801	0.708408	-0.0837	0.154881
**Frontal**	Delta	0.00036	0.473419	0.112939	0.521238	0.107777	0.073845	0.031629	-0.28954	0.101251
**Central**	Delta	0.00036	0.422017	0.104937	0.521238	0.058577	0.065313	0.307491	-0.14773	0.089553
**Parietal**	Delta	0.001172	0.354671	0.101984	0.521238	-0.05499	0.070032	0.752722	-0.03034	0.096023
**Temporal**	Delta	0.001316	0.383491	0.115633	0.521238	-0.08459	0.071412	0.364939	-0.115	0.097915

**Occipital**	Delta	0.000399	0.454222	0.117048	0.759718	0.024763	0.080715	0.364939	-0.13272	0.109739
**Prefrontal**	Theta	0.001482	0.431772	0.129169	0.789725	-0.06729	0.092243	0.923683	0.035529	0.120986
**Frontal**	Theta	0.000259	0.360171	0.090036	0.080198	0.150757	0.059736	0.143381	-0.1882	0.081906
**Central**	Theta	3.60E-05	0.44462	0.092408	0.660092	0.0767	0.062103	0.640349	-0.1067	0.085151
**Parietal**	Theta	0.000147	0.385094	0.090278	0.789725	-0.01786	0.066763	0.963004	-0.00426	0.09154
**Temporal**	Theta	0.011506	0.252291	0.097795	0.789725	-0.02488	0.063112	0.923683	-0.03464	0.086535
**Occipital**	Theta	0.000343	0.404203	0.105224	0.789725	0.040313	0.079596	0.923683	-0.06681	0.108269
**Prefrontal**	Alpha	0.000249	0.442234	0.114059	0.640156	-0.06749	0.084512	0.83237	0.045262	0.110955
**Frontal**	Alpha	2.90E-06	0.373654	0.073396	0.037716	0.165212	0.059043	0.097612	-0.19825	0.080956
**Central**	Alpha	1.40E-06	0.509437	0.090901	0.3248	0.113367	0.069887	0.83237	-0.08475	0.095824
**Parietal**	Alpha	2.50E-06	0.493907	0.094944	0.917553	0.016254	0.078506	0.83237	0.02285	0.107642
**Temporal**	Alpha	0.000468	0.322099	0.088696	0.917553	-0.00659	0.06348	0.83237	-0.02703	0.08704
**Occipital**	Alpha	1.70E-06	0.544088	0.10083	0.640156	0.070535	0.079765	0.83237	-0.0501	0.108529
**Prefrontal**	Beta	0.000129	0.488176	0.121681	0.709078	-0.06427	0.089112	0.577107	-0.06547	0.116956
**Frontal**	Beta	9.81E-05	0.344092	0.082155	0.114446	0.143753	0.068317	0.090071	-0.23227	0.093671
**Central**	Beta	1.30E-06	0.410483	0.075364	0.114446	0.130561	0.058314	0.115514	-0.16793	0.079956
**Parietal**	Beta	8.10E-06	0.356812	0.072679	0.850779	0.023048	0.061559	0.577107	-0.04803	0.084406
**Temporal**	Beta	0.000101	0.336937	0.081806	0.967606	0.002415	0.059307	0.174466	-0.1406	0.081318
**Occipital**	Beta	4.00E-07	0.494125	0.083517	0.365267	0.093013	0.069249	0.319105	-0.11829	0.09425
**Prefrontal**	Gamma	0.003746	0.459307	0.137547	0.893144	-0.0913	0.105146	0.440207	-0.12652	0.138168
**Frontal**	Gamma	0.014499	0.228831	0.091793	0.894263	0.012498	0.093768	0.440207	-0.13956	0.128568
**Central**	Gamma	0.004229	0.242655	0.079009	0.893144	0.023287	0.071172	0.440207	-0.08851	0.097587
**Parietal**	Gamma	0.010282	0.222504	0.082783	0.893144	-0.02663	0.070485	0.692994	-0.03828	0.096645
**Temporal**	Gamma	0.004229	0.326502	0.103501	0.893144	-0.04983	0.081448	0.440207	-0.1725	0.111675
**Occipital**	Gamma	0.000308	0.538371	0.126478	0.893144	0.084788	0.110475	0.440207	-0.21839	0.150417

Table S7. Linear mixed-effects model results for pairwise Pearson correlation coefficients. Fixed effects of Group, Time, and Group × Time are reported for each ROI and frequency band. The table presents regression coefficients (β), standard errors (SE), and false discovery rate–corrected p-values (p_{FDR}) (n = 48) (n = 48).

ROI1	ROI2	Band	Group p_{FDR}	Group β	Group SE	Time p_{FDR}	Time β	Time SE	Group:Time p_{FDR}	Group:Time β	Group:Time SE
**Prefrontal**	**Frontal**	**Delta**	0.299243	-0.06085	0.056827	0.752523	0.017302	0.054694	0.714695	0.026601	0.072526
**Prefrontal**	**Central**	**Delta**	0.299243	0.071079	0.067362	0.058135	0.116784	0.05563	0.409694	-0.08084	0.073286
**Prefrontal**	**Parietal**	**Delta**	0.145877	0.132969	0.077663	0.03331	0.176165	0.062253	0.126169	-0.17596	0.081927
**Prefrontal**	**Temporal**	**Delta**	0.129628	0.155531	0.072411	0.03331	0.168392	0.066962	0.161744	-0.14952	0.088622
**Prefrontal**	**Occipital**	**Delta**	0.129628	0.193811	0.087818	0.03331	0.194647	0.078812	0.126169	-0.23814	0.103358
**Frontal**	**Central**	**Delta**	0.174308	0.08114	0.052795	0.079163	0.071949	0.039037	0.454825	-0.04885	0.053525
**Frontal**	**Parietal**	**Delta**	0.145877	0.098502	0.05878	0.03331	0.121198	0.043669	0.126169	-0.11871	0.059876
**Frontal**	**Temporal**	**Delta**	0.054526	0.178026	0.05961	0.038938	0.116921	0.050686	0.126169	-0.13894	0.069497
**Frontal**	**Occipital**	**Delta**	0.076589	0.180363	0.068722	0.030721	0.166258	0.052337	0.063983	-0.20883	0.071194
**Central**	**Parietal**	**Delta**	0.139234	0.07536	0.038144	0.03331	0.073058	0.028309	0.145205	-0.07176	0.038816
**Central**	**Temporal**	**Delta**	0.139234	0.102887	0.055071	0.078591	0.090952	0.047295	0.453501	-0.06318	0.064848
**Central**	**Occipital**	**Delta**	0.139234	0.118718	0.061735	0.03331	0.113525	0.044923	0.126169	-0.12355	0.061092
**Parietal**	**Temporal**	**Delta**	0.299243	0.052915	0.050681	0.110128	0.070596	0.042831	0.681858	-0.02786	0.058726
**Parietal**	**Occipital**	**Delta**	0.145877	0.070283	0.040337	0.079163	0.05337	0.028914	0.681858	-0.0208	0.039318
**Temporal**	**Occipital**	**Delta**	0.179406	0.085101	0.057664	0.038938	0.102857	0.043826	0.161744	-0.09998	0.059615
**Prefrontal**	**Frontal**	**Theta**	0.515825	0.031738	0.044885	0.246479	0.048967	0.04196	0.540121	-0.04049	0.055561
**Prefrontal**	**Central**	**Theta**	0.177202	0.101091	0.05976	0.036538	0.118377	0.051217	0.353754	-0.0893	0.067565
**Prefrontal**	**Parietal**	**Theta**	0.177202	0.119069	0.066521	0.017885	0.167684	0.057636	0.227808	-0.14411	0.076066
**Prefrontal**	**Temporal**	**Theta**	0.094758	0.158968	0.060042	0.017885	0.18438	0.061372	0.227808	-0.15976	0.081649

**Prefrontal**	**Occipital**	**Theta**	0.177202	0.143008	0.077861	0.017885	0.203063	0.070036	0.227808	-0.19723	0.091859
**Frontal**	**Central**	**Theta**	0.094758	0.106785	0.044687	0.059541	0.074494	0.036407	0.269545	-0.07714	0.049919
**Frontal**	**Parietal**	**Theta**	0.177202	0.083738	0.049552	0.029745	0.108475	0.042139	0.227808	-0.10374	0.057778
**Frontal**	**Temporal**	**Theta**	0.094758	0.124061	0.049933	0.032365	0.11993	0.049435	0.269545	-0.10737	0.067783
**Frontal**	**Occipital**	**Theta**	0.191399	0.094947	0.061734	0.017885	0.165529	0.050136	0.227808	-0.15639	0.068227
**Central**	**Parietal**	**Theta**	0.180469	0.050193	0.030944	0.036538	0.058835	0.025692	0.353754	-0.04212	0.035227
**Central**	**Temporal**	**Theta**	0.126827	0.095773	0.044439	0.096816	0.081867	0.045836	0.456997	-0.05715	0.062847
**Central**	**Occipital**	**Theta**	0.359094	0.061137	0.057028	0.029745	0.107837	0.042778	0.353754	-0.06945	0.058185
**Parietal**	**Temporal**	**Theta**	0.359094	0.043225	0.042444	0.197703	0.054959	0.0411	0.80672	-0.01779	0.056353
**Parietal**	**Occipital**	**Theta**	0.359094	0.041025	0.039592	0.192274	0.045757	0.032812	0.918535	-0.00458	0.044658
**Temporal**	**Occipital**	**Theta**	0.569935	0.02868	0.050292	0.029745	0.099611	0.039686	0.364943	-0.06023	0.053995
**Prefrontal**	**Frontal**	**Alpha**	0.365041	0.061927	0.042208	0.103429	0.074498	0.042231	0.590062	-0.03701	0.056115
**Prefrontal**	**Central**	**Alpha**	0.365041	0.094384	0.062565	0.029606	0.134992	0.05063	0.063133	-0.14806	0.066652
**Prefrontal**	**Parietal**	**Alpha**	0.39144	0.07149	0.066731	0.029606	0.150416	0.05502	0.043006	-0.20652	0.072478
**Prefrontal**	**Temporal**	**Alpha**	0.365041	0.130869	0.059838	0.021326	0.199732	0.060556	0.043006	-0.20807	0.080517
**Prefrontal**	**Occipital**	**Alpha**	0.369592	0.105513	0.07668	0.061045	0.159758	0.069708	0.043006	-0.24058	0.091483
**Frontal**	**Central**	**Alpha**	0.365041	0.085515	0.050952	0.065694	0.086673	0.040584	0.063133	-0.12312	0.055647
**Frontal**	**Parietal**	**Alpha**	0.516616	0.040497	0.053108	0.065694	0.091072	0.042475	0.052148	-0.14113	0.058239
**Frontal**	**Temporal**	**Alpha**	0.365041	0.093598	0.051433	0.029606	0.131803	0.049371	0.065345	-0.14503	0.067694
**Frontal**	**Occipital**	**Alpha**	0.373768	0.072147	0.061528	0.029606	0.131062	0.0497	0.043006	-0.18236	0.067631
**Central**	**Parietal**	**Alpha**	0.566587	0.018527	0.03221	0.153893	0.04078	0.026925	0.239134	-0.05238	0.036917
**Central**	**Temporal**	**Alpha**	0.365041	0.068085	0.043595	0.103429	0.078892	0.044965	0.363389	-0.06894	0.061653
**Central**	**Occipital**	**Alpha**	0.373768	0.063174	0.054464	0.065694	0.091336	0.043696	0.15026	-0.10186	0.059458
**Parietal**	**Temporal**	**Alpha**	0.491142	0.035569	0.041433	0.27145	0.046117	0.041678	0.73778	-0.01919	0.057146
**Parietal**	**Occipital**	**Alpha**	0.373768	0.0506	0.039888	0.196748	0.047014	0.035083	0.701146	-0.02146	0.047769

**Temporal**	**Occipital**	**Alpha**	0.562431	0.031464	0.049295	0.065694	0.083331	0.040746	0.386456	-0.05672	0.055455
**Prefrontal**	**Frontal**	**Beta**	0.888017	0.020389	0.054729	0.134912	0.078637	0.050878	0.615807	-0.05094	0.067352
**Prefrontal**	**Central**	**Beta**	0.718378	0.050495	0.052194	0.044905	0.111036	0.043551	0.500564	-0.06736	0.057395
**Prefrontal**	**Parietal**	**Beta**	0.718378	0.066979	0.052661	0.021787	0.135091	0.043334	0.442955	-0.11065	0.05708
**Prefrontal**	**Temporal**	**Beta**	0.599945	0.096836	0.051031	0.021787	0.15972	0.052087	0.442955	-0.10939	0.069291
**Prefrontal**	**Occipital**	**Beta**	0.718378	0.075047	0.063426	0.077229	0.103309	0.053451	0.500564	-0.09498	0.069876
**Frontal**	**Central**	**Beta**	0.599945	0.066692	0.037664	0.073452	0.071533	0.034179	0.500564	-0.06036	0.046864
**Frontal**	**Parietal**	**Beta**	0.718378	0.035402	0.040393	0.044905	0.089144	0.034941	0.442955	-0.07837	0.047909
**Frontal**	**Temporal**	**Beta**	0.718378	0.059627	0.044915	0.07173	0.099994	0.044973	0.537293	-0.05894	0.061664
**Frontal**	**Occipital**	**Beta**	0.718378	0.051927	0.050116	0.044905	0.098522	0.039705	0.442955	-0.09147	0.054024
**Central**	**Parietal**	**Beta**	0.888017	0.014214	0.03018	0.07477	0.052655	0.026309	0.500564	-0.04029	0.036073
**Central**	**Temporal**	**Beta**	0.888017	0.024538	0.042941	0.123277	0.072142	0.04429	0.808993	-0.02338	0.060727
**Central**	**Occipital**	**Beta**	0.908745	0.013281	0.04914	0.07477	0.074262	0.037349	0.537293	-0.04692	0.050804
**Parietal**	**Temporal**	**Beta**	0.941695	-0.00479	0.041258	0.249847	0.048745	0.042087	0.908046	0.006684	0.057706
**Parietal**	**Occipital**	**Beta**	0.888017	0.014778	0.034494	0.102001	0.052261	0.02967	0.867663	-0.00975	0.040392
**Temporal**	**Occipital**	**Beta**	0.941695	-0.00349	0.047564	0.073452	0.082116	0.038457	0.648464	-0.0339	0.052332
**Prefrontal**	**Frontal**	**Gamma**	0.737917	-0.0257	0.063937	0.522639	0.036957	0.055705	0.786318	-0.03606	0.073534
**Prefrontal**	**Central**	**Gamma**	0.887478	-0.00845	0.059533	0.297623	0.067982	0.047813	0.786318	-0.04491	0.062927
**Prefrontal**	**Parietal**	**Gamma**	0.473524	0.053219	0.06433	0.446392	0.05151	0.05035	0.786318	-0.04397	0.066211
**Prefrontal**	**Temporal**	**Gamma**	0.216787	0.096845	0.060921	0.29113	0.11209	0.056358	0.786318	-0.08245	0.074589
**Prefrontal**	**Occipital**	**Gamma**	0.452099	0.06817	0.074326	0.522639	0.036876	0.057442	0.786318	-0.02612	0.074819
**Frontal**	**Central**	**Gamma**	0.092086	0.087738	0.042191	0.29475	0.061955	0.041349	0.786318	-0.05685	0.056695
**Frontal**	**Parietal**	**Gamma**	0.092086	0.111396	0.046729	0.446392	0.039967	0.040583	0.786318	-0.04477	0.055645
**Frontal**	**Temporal**	**Gamma**	0.092086	0.102147	0.049278	0.29475	0.072831	0.047124	0.786318	-0.03888	0.064613
**Frontal**	**Occipital**	**Gamma**	0.092086	0.140585	0.056279	0.29113	0.078589	0.041676	0.786318	-0.07787	0.056682

**Central**	**Parietal**	**Gamma**	0.092086	0.074246	0.033008	0.494155	0.024986	0.029255	0.786318	-0.02217	0.040112
**Central**	**Temporal**	**Gamma**	0.340038	0.060072	0.046951	0.446392	0.047664	0.04834	0.943338	-0.00472	0.06628
**Central**	**Occipital**	**Gamma**	0.092086	0.106852	0.052038	0.29475	0.061835	0.040309	0.786318	-0.0435	0.054837
**Parietal**	**Temporal**	**Gamma**	0.452099	0.039342	0.042182	0.522639	0.031724	0.043507	0.786318	0.020342	0.059654
**Parietal**	**Occipital**	**Gamma**	0.092086	0.088056	0.034807	0.29113	0.056613	0.030548	0.786318	-0.029	0.041594
**Temporal**	**Occipital**	**Gamma**	0.452099	0.049672	0.050733	0.29113	0.078614	0.044036	0.786318	-0.02305	0.059955
**Prefrontal**	**Frontal**	**Broad**	0.313165	-0.0621	0.0561	0.602282	0.027981	0.053493	0.826233	0.021615	0.070901
**Prefrontal**	**Central**	**Broad**	0.515654	0.044979	0.063586	0.055954	0.111006	0.051339	0.436525	-0.0718	0.06758
**Prefrontal**	**Parietal**	**Broad**	0.287181	0.101202	0.072498	0.055954	0.155882	0.056977	0.206067	-0.15171	0.074936
**Prefrontal**	**Temporal**	**Broad**	0.192576	0.137138	0.069388	0.055954	0.159319	0.064523	0.228528	-0.13346	0.085416
**Prefrontal**	**Occipital**	**Broad**	0.192576	0.165215	0.081858	0.055954	0.163715	0.070859	0.206067	-0.19939	0.092754
**Frontal**	**Central**	**Broad**	0.294652	0.061724	0.049547	0.109237	0.06673	0.038613	0.531806	-0.04573	0.052944
**Frontal**	**Parietal**	**Broad**	0.287181	0.075816	0.055109	0.055954	0.102485	0.042164	0.226202	-0.09893	0.057813
**Frontal**	**Temporal**	**Broad**	0.081165	0.160821	0.056418	0.055954	0.107376	0.049752	0.206067	-0.12613	0.068217
**Frontal**	**Occipital**	**Broad**	0.097215	0.16332	0.064404	0.055954	0.144444	0.049278	0.097858	-0.18675	0.067034
**Central**	**Parietal**	**Broad**	0.26465	0.05813	0.035587	0.055954	0.059301	0.027031	0.228528	-0.05798	0.037062
**Central**	**Temporal**	**Broad**	0.287181	0.075995	0.052067	0.115118	0.077371	0.046522	0.656521	-0.04068	0.063788
**Central**	**Occipital**	**Broad**	0.26465	0.097478	0.058342	0.055954	0.096163	0.04206	0.206067	-0.1054	0.057197
**Parietal**	**Temporal**	**Broad**	0.612166	0.024667	0.048484	0.217448	0.05428	0.042323	0.954858	0.003294	0.05803
**Parietal**	**Occipital**	**Broad**	0.294652	0.048726	0.039056	0.109237	0.050467	0.028915	0.826233	-0.01147	0.039326
**Temporal**	**Occipital**	**Broad**	0.308122	0.063659	0.054569	0.066762	0.084763	0.041589	0.375021	-0.06912	0.056574

Table S8. Linear mixed-effects model results for phase transfer entropy. Fixed effects of Group, Time, and Group × Time are reported for each ROI and frequency band. The table presents regression

coefficients (β), standard errors (SE), and false discovery rate–corrected p-values (p_{FDR}) (n = 48) (n = 48).

ROI1	ROI2	Band	Group p_{FDR}	Group β	Group SE	Time p_{FDR}	Time β	Time SE	Group:Time p_{FDR}	Group:Time β	Group:Time SE
**Prefrontal**	**Frontal**	**Delta**	0.987068	-0.04279	0.087021	0.14739	-0.18907	0.087469	0.208491	0.228094	0.116256
**Prefrontal**	**Central**	**Delta**	0.987068	-0.01494	0.093558	0.14739	-0.19739	0.092908	0.208491	0.200781	0.123402
**Prefrontal**	**Parietal**	**Delta**	0.987068	0.001468	0.090291	0.14739	-0.16805	0.095127	0.208491	0.178054	0.126788
**Prefrontal**	**Temporal**	**Delta**	0.987068	-0.09505	0.089686	0.188567	-0.141	0.09575	0.208491	0.217876	0.127725
**Prefrontal**	**Occipital**	**Delta**	0.987068	-0.01165	0.090491	0.14739	-0.16346	0.096307	0.208491	0.188503	0.127749
**Frontal**	**Prefrontal**	**Delta**	0.987068	-0.03007	0.086185	0.14739	-0.16532	0.088187	0.208491	0.204928	0.117331
**Frontal**	**Central**	**Delta**	0.987068	-0.0314	0.084024	0.14739	-0.15844	0.083614	0.208491	0.170353	0.114645
**Frontal**	**Parietal**	**Delta**	0.987068	-0.00516	0.083953	0.16685	-0.13483	0.085752	0.234177	0.145679	0.117577
**Frontal**	**Temporal**	**Delta**	0.987068	-0.11421	0.084582	0.198852	-0.12203	0.085941	0.208491	0.20509	0.117836
**Frontal**	**Occipital**	**Delta**	0.987068	-0.02986	0.085483	0.14739	-0.15241	0.085757	0.208491	0.17713	0.116908
**Central**	**Prefrontal**	**Delta**	0.987068	-0.06293	0.089455	0.14739	-0.15708	0.09021	0.208491	0.224261	0.119922
**Central**	**Frontal**	**Delta**	0.987068	-0.09315	0.081318	0.14739	-0.14267	0.080581	0.208491	0.217975	0.110487
**Central**	**Parietal**	**Delta**	0.987068	-0.01704	0.084394	0.14739	-0.14925	0.074089	0.208491	0.145585	0.101586
**Central**	**Temporal**	**Delta**	0.987068	-0.09832	0.085043	0.14739	-0.15414	0.084625	0.208491	0.210677	0.116032
**Central**	**Occipital**	**Delta**	0.987068	-0.03132	0.088087	0.14739	-0.17561	0.078985	0.208491	0.18468	0.107564
**Parietal**	**Prefrontal**	**Delta**	0.987068	-0.03189	0.089652	0.25275	-0.11471	0.094355	0.208491	0.194534	0.125752
**Parietal**	**Frontal**	**Delta**	0.987068	-0.0519	0.083202	0.232606	-0.10983	0.084209	0.208491	0.190781	0.115462
**Parietal**	**Central**	**Delta**	0.987068	-0.00145	0.085829	0.14739	-0.14051	0.074481	0.208491	0.143375	0.102123
**Parietal**	**Temporal**	**Delta**	0.987068	-0.06268	0.085701	0.16685	-0.13623	0.087345	0.208491	0.176317	0.119761

**Parietal**	**Occipital**	**Delta**	0.987068	-0.02415	0.089297	0.14739	-0.17371	0.076387	0.208491	0.140277	0.103988
**Temporal**	**Prefrontal**	**Delta**	0.987068	-0.05315	0.088795	0.316529	-0.09763	0.094799	0.208491	0.178007	0.126457
**Temporal**	**Frontal**	**Delta**	0.987068	-0.0894	0.083292	0.232606	-0.10817	0.084082	0.208491	0.194155	0.115287
**Temporal**	**Central**	**Delta**	0.987068	-0.01286	0.08521	0.14739	-0.15757	0.084411	0.208491	0.154101	0.115738
**Temporal**	**Parietal**	**Delta**	0.987068	0.007645	0.085136	0.14739	-0.14869	0.087446	0.323497	0.121728	0.1199
**Temporal**	**Occipital**	**Delta**	0.987068	-0.03061	0.086994	0.14739	-0.17667	0.082281	0.208491	0.18687	0.112105
**Occipital**	**Prefrontal**	**Delta**	0.987068	-0.01534	0.089964	0.369413	-0.08653	0.095889	0.208491	0.171316	0.12721
**Occipital**	**Frontal**	**Delta**	0.987068	-0.0453	0.084466	0.278573	-0.09647	0.085101	0.208491	0.180953	0.116019
**Occipital**	**Central**	**Delta**	0.987068	0.015753	0.08823	0.14739	-0.13657	0.078934	0.208491	0.142728	0.107493
**Occipital**	**Parietal**	**Delta**	0.987068	0.006896	0.08868	0.14739	-0.14345	0.076243	0.335407	0.100528	0.103795
**Occipital**	**Temporal**	**Delta**	0.987068	-0.06925	0.087386	0.162983	-0.1348	0.083184	0.208491	0.200353	0.113342
**Prefrontal**	**Frontal**	**Theta**	0.999627	-0.04936	0.069449	0.051917	-0.20218	0.071377	0.242226	0.183672	0.09499
**Prefrontal**	**Central**	**Theta**	0.999627	-0.02749	0.071049	0.051917	-0.18252	0.073298	0.242226	0.178825	0.097569
**Prefrontal**	**Parietal**	**Theta**	0.999627	0.017092	0.069732	0.051917	-0.16349	0.073869	0.242226	0.171421	0.098489
**Prefrontal**	**Temporal**	**Theta**	0.999627	0.01954	0.066003	0.063504	-0.14528	0.070482	0.242226	0.135648	0.094022
**Prefrontal**	**Occipital**	**Theta**	0.999627	0.059601	0.068373	0.094863	-0.12559	0.072885	0.242226	0.128947	0.096692
**Frontal**	**Prefrontal**	**Theta**	0.999627	-0.04655	0.069213	0.051917	-0.20793	0.070675	0.242226	0.17804	0.094021
**Frontal**	**Central**	**Theta**	0.999627	-0.03013	0.060772	0.053042	-0.13526	0.062681	0.242226	0.151158	0.085944
**Frontal**	**Parietal**	**Theta**	0.999627	0.030831	0.064034	0.069919	-0.12514	0.066022	0.242226	0.138608	0.090524
**Frontal**	**Temporal**	**Theta**	0.999627	0.014826	0.061262	0.069919	-0.12251	0.063016	0.242226	0.113161	0.086403
**Frontal**	**Occipital**	**Theta**	0.999627	0.065667	0.06441	0.11371	-0.1025	0.064167	0.242226	0.10815	0.08747

**Central**	**Prefrontal**	**Theta**	0.999627	-0.03899	0.069642	0.051917	-0.19791	0.072511	0.242226	0.171978	0.096575
**Central**	**Frontal**	**Theta**	0.999627	-0.04322	0.059979	0.051917	-0.14177	0.061863	0.242226	0.147713	0.084823
**Central**	**Parietal**	**Theta**	0.999627	0.003301	0.062168	0.051917	-0.13013	0.0535	0.242226	0.125318	0.073356
**Central**	**Temporal**	**Theta**	0.999627	-2.83E-05	0.060445	0.051917	-0.15109	0.062344	0.242226	0.134431	0.085482
**Central**	**Occipital**	**Theta**	0.999627	0.043373	0.066157	0.051917	-0.13617	0.055362	0.242226	0.127246	0.075355
**Parietal**	**Prefrontal**	**Theta**	0.999627	0.004018	0.067572	0.051917	-0.16953	0.072158	0.242226	0.149023	0.096257
**Parietal**	**Frontal**	**Theta**	0.999627	0.017453	0.062287	0.069919	-0.12097	0.064245	0.242226	0.118194	0.088088
**Parietal**	**Central**	**Theta**	0.999627	0.001973	0.061703	0.051917	-0.11876	0.054242	0.242226	0.110653	0.074372
**Parietal**	**Temporal**	**Theta**	0.999627	0.029864	0.061349	0.067247	-0.1261	0.063277	0.28083	0.094134	0.086761
**Parietal**	**Occipital**	**Theta**	0.999627	0.032048	0.066857	0.051917	-0.12803	0.053456	0.242226	0.088664	0.072737
**Temporal**	**Prefrontal**	**Theta**	0.999627	0.009537	0.065042	0.051917	-0.15346	0.069456	0.242226	0.128387	0.092653
**Temporal**	**Frontal**	**Theta**	0.999627	0.005068	0.060652	0.069919	-0.12067	0.062558	0.242226	0.108167	0.085775
**Temporal**	**Central**	**Theta**	0.999627	0.005113	0.060837	0.051917	-0.1397	0.062749	0.242226	0.13025	0.086037
**Temporal**	**Parietal**	**Theta**	0.999627	0.034893	0.062411	0.067247	-0.1283	0.064372	0.242226	0.108217	0.088262
**Temporal**	**Occipital**	**Theta**	0.999627	0.052512	0.064283	0.051917	-0.13263	0.057591	0.242226	0.117354	0.078429
**Occipital**	**Prefrontal**	**Theta**	0.999627	0.042612	0.066564	0.069919	-0.13579	0.070957	0.242226	0.119819	0.094135
**Occipital**	**Frontal**	**Theta**	0.999627	0.046936	0.062637	0.103716	-0.10722	0.064557	0.248332	0.104121	0.088033
**Occipital**	**Central**	**Theta**	0.999627	0.040991	0.065614	0.051917	-0.12965	0.056241	0.242226	0.121943	0.076564
**Occipital**	**Parietal**	**Theta**	0.999627	0.026764	0.066915	0.051917	-0.13561	0.05429	0.242226	0.102571	0.073879
**Occipital**	**Temporal**	**Theta**	0.999627	0.043387	0.062743	0.051917	-0.13946	0.057485	0.242226	0.118096	0.0783
**Prefrontal**	**Frontal**	**Alpha**	0.921908	-0.02205	0.053902	0.034719	-0.17462	0.054305	0.304282	0.120876	0.072187
**Prefrontal**	**Central**	**Alpha**	0.921908	0.008554	0.049976	0.034719	-0.14681	0.051546	0.304282	0.11308	0.068614

**Prefrontal**	**Parietal**	**Alpha**	0.921908	0.02454	0.051797	0.04655	-0.11231	0.053941	0.304282	0.091391	0.071842
**Prefrontal**	**Temporal**	**Alpha**	0.921908	0.047311	0.048855	0.044738	-0.11073	0.051206	0.304282	0.082002	0.068227
**Prefrontal**	**Occipital**	**Alpha**	0.921908	0.039894	0.054383	0.17529	-0.07739	0.056618	0.326615	0.079178	0.074973
**Frontal**	**Prefrontal**	**Alpha**	0.921908	-0.02277	0.055529	0.034719	-0.16731	0.057449	0.304282	0.108062	0.076484
**Frontal**	**Central**	**Alpha**	0.931527	-0.00388	0.045001	0.035183	-0.12084	0.046269	0.304282	0.103965	0.063441
**Frontal**	**Parietal**	**Alpha**	0.921908	0.025276	0.049226	0.04655	-0.1018	0.048658	0.304282	0.080604	0.066716
**Frontal**	**Temporal**	**Alpha**	0.921908	0.036165	0.046379	0.044738	-0.10207	0.045383	0.304282	0.073717	0.062225
**Frontal**	**Occipital**	**Alpha**	0.921908	0.037266	0.051376	0.155229	-0.07307	0.050325	0.316051	0.075509	0.068589
**Central**	**Prefrontal**	**Alpha**	0.921908	0.016599	0.053495	0.034719	-0.14854	0.055376	0.304282	0.1368	0.073727
**Central**	**Frontal**	**Alpha**	0.921908	0.006246	0.045523	0.034719	-0.12697	0.046804	0.304282	0.14007	0.064175
**Central**	**Parietal**	**Alpha**	0.921908	0.009997	0.048171	0.036689	-0.10161	0.040192	0.304282	0.078453	0.055109
**Central**	**Temporal**	**Alpha**	0.921908	0.017576	0.04679	0.034719	-0.12511	0.047039	0.304282	0.088999	0.064497
**Central**	**Occipital**	**Alpha**	0.921908	0.021559	0.052186	0.038984	-0.1026	0.043407	0.304282	0.101875	0.05908
**Parietal**	**Prefrontal**	**Alpha**	0.921908	0.045072	0.051596	0.044738	-0.12475	0.055097	0.304282	0.118273	0.073499
**Parietal**	**Frontal**	**Alpha**	0.921908	0.052027	0.047126	0.04655	-0.10017	0.04789	0.304282	0.101474	0.065664
**Parietal**	**Central**	**Alpha**	0.921908	0.023511	0.047068	0.036689	-0.09593	0.038837	0.304282	0.064909	0.053251
**Parietal**	**Temporal**	**Alpha**	0.921908	0.030763	0.047214	0.044738	-0.10701	0.048698	0.348945	0.065504	0.066771
**Parietal**	**Occipital**	**Alpha**	0.921908	0.012339	0.054193	0.044738	-0.09812	0.045647	0.304282	0.076386	0.062134
**Temporal**	**Prefrontal**	**Alpha**	0.921908	0.046401	0.050595	0.044738	-0.11739	0.054029	0.304282	0.093029	0.072074
**Temporal**	**Frontal**	**Alpha**	0.921908	0.039552	0.04606	0.036689	-0.10856	0.044691	0.304282	0.092251	0.061277
**Temporal**	**Central**	**Alpha**	0.921908	0.009895	0.049402	0.034719	-0.12774	0.046517	0.304282	0.074889	0.063781

**Temporal**	**Parietal**	**Alpha**	0.921908	0.009152	0.050605	0.038984	-0.11477	0.048773	0.348945	0.062966	0.066875
**Temporal**	**Occipital**	**Alpha**	0.921908	0.022237	0.050608	0.044738	-0.10177	0.045862	0.304282	0.086758	0.062462
**Occipital**	**Prefrontal**	**Alpha**	0.921908	0.064928	0.052502	0.082095	-0.10033	0.055968	0.316051	0.082468	0.074249
**Occipital**	**Frontal**	**Alpha**	0.921908	0.066067	0.048433	0.06924	-0.09087	0.048134	0.304282	0.080158	0.065613
**Occipital**	**Central**	**Alpha**	0.921908	0.039265	0.050677	0.034719	-0.11475	0.04166	0.304282	0.071097	0.056697
**Occipital**	**Parietal**	**Alpha**	0.921908	0.016128	0.053565	0.036689	-0.11244	0.045916	0.348945	0.059046	0.062508
**Occipital**	**Temporal**	**Alpha**	0.921908	0.048512	0.047858	0.036689	-0.11129	0.044849	0.304282	0.07206	0.0611
**Prefrontal**	**Frontal**	**Beta**	0.990589	-0.04504	0.078673	0.023782	-0.21822	0.080244	0.217511	0.210825	0.106743
**Prefrontal**	**Central**	**Beta**	0.990589	-0.05424	0.071559	0.018876	-0.21216	0.072176	0.217511	0.192915	0.095949
**Prefrontal**	**Parietal**	**Beta**	0.990589	-0.03842	0.069042	0.028938	-0.17702	0.072297	0.217511	0.159965	0.096324
**Prefrontal**	**Temporal**	**Beta**	0.990589	-0.00113	0.069907	0.049977	-0.14948	0.07287	0.266816	0.118214	0.09706
**Prefrontal**	**Occipital**	**Beta**	0.990589	0.027754	0.074517	0.143708	-0.11458	0.077635	0.405915	0.088388	0.102808
**Frontal**	**Prefrontal**	**Beta**	0.990589	-0.02947	0.079154	0.024377	-0.21036	0.080452	0.217511	0.18704	0.106998
**Frontal**	**Central**	**Beta**	0.990589	-0.05493	0.061781	0.018876	-0.18324	0.063722	0.217511	0.176285	0.087371
**Frontal**	**Parietal**	**Beta**	0.990589	-0.02626	0.063689	0.029813	-0.15832	0.065636	0.217511	0.144646	0.089996
**Frontal**	**Temporal**	**Beta**	0.990589	0.00519	0.065053	0.033397	-0.14754	0.065148	0.266816	0.111849	0.089326
**Frontal**	**Occipital**	**Beta**	0.990589	0.027753	0.069413	0.089085	-0.1192	0.068019	0.336753	0.093814	0.092705
**Central**	**Prefrontal**	**Beta**	0.990589	-0.01547	0.07321	0.028938	-0.18182	0.072792	0.217511	0.160039	0.096691
**Central**	**Frontal**	**Beta**	0.990589	-0.03638	0.063017	0.024377	-0.17216	0.064997	0.217511	0.178532	0.08912
**Central**	**Parietal**	**Beta**	0.990589	-0.02346	0.063159	0.018876	-0.15538	0.051676	0.217511	0.137254	0.070855
**Central**	**Temporal**	**Beta**	0.990589	-0.00079	0.06497	0.023782	-0.17543	0.064074	0.242165	0.13034	0.087854

**Central**	**Occipital**	**Beta**	0.990589	0.034776	0.07118	0.033397	-0.12908	0.057291	0.266816	0.093781	0.077959
**Parietal**	**Prefrontal**	**Beta**	0.990589	0.006643	0.070776	0.044155	-0.15694	0.073977	0.242165	0.144852	0.09855
**Parietal**	**Frontal**	**Beta**	0.990589	-0.00344	0.064726	0.031279	-0.15837	0.06676	0.217511	0.16612	0.091536
**Parietal**	**Central**	**Beta**	0.990589	-0.01992	0.062987	0.018876	-0.16525	0.052846	0.217511	0.154139	0.072459
**Parietal**	**Temporal**	**Beta**	0.990589	0.013727	0.064393	0.028938	-0.1634	0.066416	0.266816	0.108414	0.091065
**Parietal**	**Occipital**	**Beta**	0.990589	0.042243	0.071273	0.032558	-0.13064	0.056802	0.433555	0.060802	0.077289
**Temporal**	**Prefrontal**	**Beta**	0.990589	0.016204	0.070039	0.065522	-0.14001	0.073144	0.266816	0.117045	0.097436
**Temporal**	**Frontal**	**Beta**	0.990589	0.000768	0.064946	0.031589	-0.15541	0.066185	0.217511	0.144023	0.090748
**Temporal**	**Central**	**Beta**	0.990589	-0.02432	0.063275	0.018876	-0.1944	0.06386	0.217511	0.159366	0.08756
**Temporal**	**Parietal**	**Beta**	0.990589	-0.01413	0.063363	0.024377	-0.17232	0.065354	0.266816	0.120273	0.089609
**Temporal**	**Occipital**	**Beta**	0.990589	0.038779	0.067687	0.028938	-0.14621	0.058619	0.266816	0.096966	0.079807
**Occipital**	**Prefrontal**	**Beta**	0.990589	0.02519	0.071327	0.092037	-0.12865	0.074759	0.266816	0.117175	0.099046
**Occipital**	**Frontal**	**Beta**	0.990589	0.002972	0.066283	0.032558	-0.15146	0.065894	0.217511	0.156772	0.089822
**Occipital**	**Central**	**Beta**	0.990589	-0.00646	0.066615	0.018876	-0.16956	0.054235	0.217511	0.150115	0.073806
**Occipital**	**Parietal**	**Beta**	0.990589	0.001754	0.066871	0.018876	-0.15568	0.053388	0.266816	0.094881	0.072644
**Occipital**	**Temporal**	**Beta**	0.990589	0.023683	0.064636	0.018876	-0.16695	0.056091	0.217511	0.123765	0.076367
**Prefrontal**	**Frontal**	**Gamma**	0.102975	-0.18093	0.109763	0.496456	-0.15425	0.104116	0.426429	0.194044	0.137962
**Prefrontal**	**Central**	**Gamma**	0.074563	-0.20035	0.107013	0.496456	-0.13431	0.093895	0.426429	0.193381	0.123983
**Prefrontal**	**Parietal**	**Gamma**	0.072903	-0.20418	0.10743	0.496456	-0.098	0.097451	0.426429	0.160765	0.12886
**Prefrontal**	**Temporal**	**Gamma**	0.055193	-0.21773	0.104644	0.496456	-0.09913	0.098194	0.426429	0.156388	0.130046
**Prefrontal**	**Occipital**	**Gamma**	0.097971	-0.1861	0.108994	0.512758	-0.0726	0.096146	0.426429	0.131487	0.125974
**Frontal**	**Prefrontal**	**Gamma**	0.102093	-0.18616	0.111506	0.496456	-0.16207	0.104256	0.426429	0.205755	0.138051

**Frontal**	**Central**	**Gamma**	0.041897	-0.26696	0.100526	0.496456	-0.10474	0.091856	0.426429	0.167306	0.125946
**Frontal**	**Parietal**	**Gamma**	0.041897	-0.2514	0.10312	0.512758	-0.06725	0.090932	0.426429	0.135747	0.12468
**Frontal**	**Temporal**	**Gamma**	0.041897	-0.25287	0.100357	0.496456	-0.0827	0.089588	0.426429	0.131527	0.122836
**Frontal**	**Occipital**	**Gamma**	0.045035	-0.24352	0.106057	0.500214	-0.07298	0.089472	0.426429	0.138866	0.121788
**Central**	**Prefrontal**	**Gamma**	0.07551	-0.20249	0.109524	0.496456	-0.13916	0.095254	0.426429	0.188409	0.125732
**Central**	**Frontal**	**Gamma**	0.041897	-0.26653	0.101869	0.496456	-0.09821	0.093595	0.426429	0.147444	0.128331
**Central**	**Parietal**	**Gamma**	0.04851	-0.23001	0.103723	0.682311	-0.03352	0.081622	0.476317	0.080045	0.111914
**Central**	**Temporal**	**Gamma**	0.041897	-0.25097	0.098728	0.496456	-0.09947	0.088631	0.426429	0.135892	0.121525
**Central**	**Occipital**	**Gamma**	0.055193	-0.23094	0.110758	0.560439	-0.05032	0.082148	0.44341	0.096958	0.111728
**Parietal**	**Prefrontal**	**Gamma**	0.055193	-0.22512	0.10806	0.496456	-0.11964	0.097384	0.426429	0.166748	0.128735
**Parietal**	**Frontal**	**Gamma**	0.041897	-0.2671	0.102187	0.496456	-0.07738	0.09095	0.426429	0.126605	0.124705
**Parietal**	**Central**	**Gamma**	0.041897	-0.24509	0.102024	0.560439	-0.04964	0.079696	0.44341	0.090519	0.109274
**Parietal**	**Temporal**	**Gamma**	0.041897	-0.2557	0.095872	0.496456	-0.1079	0.088194	0.426429	0.132155	0.120926
**Parietal**	**Occipital**	**Gamma**	0.041897	-0.25155	0.106162	0.496456	-0.08204	0.079577	0.44341	0.088862	0.108238
**Temporal**	**Prefrontal**	**Gamma**	0.060387	-0.21008	0.103885	0.496456	-0.10929	0.097594	0.426429	0.145051	0.129258
**Temporal**	**Frontal**	**Gamma**	0.041897	-0.23439	0.097354	0.496456	-0.07984	0.087982	0.44341	0.102937	0.120634
**Temporal**	**Central**	**Gamma**	0.041897	-0.23224	0.094651	0.496456	-0.10263	0.085165	0.426429	0.126809	0.116773
**Temporal**	**Parietal**	**Gamma**	0.041897	-0.22079	0.093759	0.496456	-0.09401	0.085817	0.431163	0.111719	0.117666
**Temporal**	**Occipital**	**Gamma**	0.049636	-0.22241	0.101739	0.496456	-0.11316	0.081318	0.426429	0.14263	0.110648
**Occipital**	**Prefrontal**	**Gamma**	0.072903	-0.20167	0.105831	0.496456	-0.09778	0.093575	0.426429	0.134408	0.122621
**Occipital**	**Frontal**	**Gamma**	0.041897	-0.24985	0.100887	0.496456	-0.08416	0.086829	0.426429	0.123813	0.118209

**Occipital**	**Central**	**Gamma**	0.045441	-0.23738	0.104664	0.496456	-0.06797	0.077439	0.431163	0.101895	0.105323
**Occipital**	**Parietal**	**Gamma**	0.041897	-0.24116	0.102593	0.496456	-0.08201	0.076817	0.446343	0.082571	0.104483
**Occipital**	**Temporal**	**Gamma**	0.041897	-0.24994	0.100102	0.496456	-0.13032	0.080769	0.426429	0.15953	0.109909
**Prefrontal**	**Frontal**	**Broad**	0.904559	-0.1536	0.116036	0.422567	-0.21606	0.111693	0.29366	0.316119	0.14811
**Prefrontal**	**Central**	**Broad**	0.904559	-0.03772	0.10934	0.422567	-0.19741	0.106782	0.29366	0.251536	0.141703
**Prefrontal**	**Parietal**	**Broad**	0.923867	0.022438	0.105114	0.422567	-0.15565	0.109341	0.29366	0.20582	0.145619
**Prefrontal**	**Temporal**	**Broad**	0.904559	-0.10458	0.115809	0.423203	-0.11255	0.119084	0.29366	0.218984	0.158485
**Prefrontal**	**Occipital**	**Broad**	0.904559	0.062162	0.111963	0.422567	-0.12228	0.115093	0.29366	0.191	0.152257
**Frontal**	**Prefrontal**	**Broad**	0.904559	-0.07017	0.121092	0.422567	-0.1818	0.122123	0.29366	0.253557	0.162347
**Frontal**	**Central**	**Broad**	0.904559	-0.05918	0.099349	0.422567	-0.14277	0.0998	0.29366	0.211777	0.136839
**Frontal**	**Parietal**	**Broad**	0.947225	0.014284	0.097693	0.423203	-0.09878	0.100763	0.315576	0.155626	0.138159
**Frontal**	**Temporal**	**Broad**	0.904559	-0.12312	0.109363	0.471157	-0.0772	0.106684	0.29366	0.19307	0.146277
**Frontal**	**Occipital**	**Broad**	0.904559	0.04387	0.105867	0.43824	-0.08294	0.103179	0.315576	0.161528	0.140617
**Central**	**Prefrontal**	**Broad**	0.904559	-0.09223	0.121969	0.422567	-0.17717	0.117636	0.29366	0.274797	0.156007
**Central**	**Frontal**	**Broad**	0.904559	-0.1956	0.109314	0.422567	-0.14851	0.100807	0.29366	0.289477	0.138219
**Central**	**Parietal**	**Broad**	0.966987	0.004033	0.097169	0.422567	-0.1078	0.085536	0.29366	0.145801	0.117281
**Central**	**Temporal**	**Broad**	0.904559	-0.08416	0.110339	0.422567	-0.11579	0.10478	0.29366	0.181849	0.143666
**Central**	**Occipital**	**Broad**	0.904559	0.05723	0.108001	0.422567	-0.10352	0.09155	0.29366	0.160132	0.124621
**Parietal**	**Prefrontal**	**Broad**	0.904559	-0.06198	0.121109	0.422567	-0.13648	0.122479	0.29366	0.247686	0.162846
**Parietal**	**Frontal**	**Broad**	0.904559	-0.1562	0.110454	0.422567	-0.11496	0.105061	0.29366	0.26228	0.144052
**Parietal**	**Central**	**Broad**	0.922485	-0.02561	0.100542	0.422567	-0.11674	0.086528	0.29366	0.170572	0.118642
**Parietal**	**Temporal**	**Broad**	0.904559	-0.04223	0.11054	0.429594	-0.09377	0.107817	0.430799	0.128313	0.147831

**Parietal**	**Occipital**	**Broad**	0.904559	0.071334	0.104913	0.422567	-0.10452	0.086759	0.45065	0.09554	0.118079
**Temporal**	**Prefrontal**	**Broad**	0.904559	-0.09707	0.120692	0.429594	-0.10551	0.124987	0.29366	0.224554	0.166411
**Temporal**	**Frontal**	**Broad**	0.904559	-0.20673	0.111318	0.422567	-0.10899	0.104776	0.29366	0.265703	0.143662
**Temporal**	**Central**	**Broad**	0.904559	-0.03165	0.100616	0.422567	-0.14116	0.10052	0.29366	0.17671	0.137826
**Temporal**	**Parietal**	**Broad**	0.904559	0.04101	0.099548	0.422567	-0.10815	0.102676	0.498226	0.095737	0.140782
**Temporal**	**Occipital**	**Broad**	0.904559	0.055612	0.107852	0.422567	-0.10728	0.097945	0.320066	0.145791	0.1334
**Occipital**	**Prefrontal**	**Broad**	0.904559	-0.05517	0.11984	0.423203	-0.11221	0.123656	0.29366	0.234597	0.163632
**Occipital**	**Frontal**	**Broad**	0.904559	-0.16113	0.111331	0.422567	-0.10776	0.105515	0.29366	0.269102	0.143764
**Occipital**	**Central**	**Broad**	0.966987	-0.0049	0.103581	0.422567	-0.1188	0.091413	0.29366	0.1847	0.124474
**Occipital**	**Parietal**	**Broad**	0.904559	0.043872	0.098825	0.422567	-0.10646	0.086564	0.45854	0.090772	0.117864
**Occipital**	**Temporal**	**Broad**	0.904559	-0.05339	0.113243	0.423203	-0.09309	0.102154	0.29366	0.169933	0.139124

Table S9. Linear mixed-effects model results for phase lag index. Fixed effects of Group, Time, and Group × Time are reported for each ROI and frequency band. The table presents regression coefficients (β), standard errors (SE), and false discovery rate–corrected p-values (p_{FDR}) (n = 48).

ROI1	ROI2	Band	Group p_{FDR}	Group β	Group SE	Time p_{FDR}	Time β	Time SE	GroupTime p_{FDR}	GroupTime β	GroupTime SE
**Prefrontal**	**Frontal**	**Delta**	0.880709	-0.00304	0.005417	0.947012	-0.00478	0.006298	0.859363	-0.00161	0.007661
**Prefrontal**	**Central**	**Delta**	0.538337	0.010877	0.008018	0.692727	0.013634	0.009095	0.467385	-0.01893	0.010805
**Prefrontal**	**Parietal**	**Delta**	0.880709	0.00495	0.010486	0.974219	-0.00054	0.012394	0.859363	0.005517	0.014554
**Prefrontal**	**Temporal**	**Delta**	0.665638	0.006815	0.006672	0.974219	-0.00026	0.007938	0.859363	-0.00585	0.009669
**Prefrontal**	**Occipital**	**Delta**	0.895033	0.001192	0.009001	0.974219	-0.00192	0.010184	0.859363	-0.00579	0.012787
**Frontal**	**Central**	**Delta**	0.665638	0.007326	0.006765	0.974219	0.001299	0.007524	0.859363	-0.00635	0.008754

**Frontal**	**Parietal**	**Delta**	0.880709	0.003826	0.008412	0.947012	0.005349	0.009355	0.859363	-0.00581	0.010885
**Frontal**	**Temporal**	**Delta**	0.880709	0.002673	0.005267	0.90865	-0.0058	0.005933	0.859363	0.001314	0.007387
**Frontal**	**Occipital**	**Delta**	0.895033	0.001288	0.006843	0.947012	-0.00267	0.006784	0.859363	-0.0031	0.008558
**Central**	**Parietal**	**Delta**	0.895033	-0.00133	0.005738	0.90865	0.005901	0.00645	0.859363	0.00521	0.007773
**Central**	**Temporal**	**Delta**	0.078911	0.016759	0.005809	0.099827	0.017752	0.006338	0.145579	-0.02008	0.007541
**Central**	**Occipital**	**Delta**	0.880709	-0.0027	0.00708	0.947012	0.004602	0.007207	0.859363	-0.00159	0.008682
**Parietal**	**Temporal**	**Delta**	0.349215	0.011118	0.006526	0.947012	0.003407	0.007201	0.859363	-0.00656	0.008528
**Parietal**	**Occipital**	**Delta**	0.335796	0.014041	0.007552	0.737477	0.010551	0.008095	0.859363	-0.00949	0.009784
**Temporal**	**Occipital**	**Delta**	0.191114	0.013096	0.005752	0.538198	0.010026	0.005494	0.467385	-0.01213	0.007144
**Prefrontal**	**Frontal**	**Theta**	0.877838	-0.00339	0.005851	0.737342	-0.00563	0.005914	0.856857	0.003428	0.00706
**Prefrontal**	**Central**	**Theta**	0.950258	0.002345	0.007653	0.737342	0.008815	0.008561	0.856857	-0.00881	0.01014
**Prefrontal**	**Parietal**	**Theta**	0.634585	0.009831	0.007792	0.98959	0.000121	0.009209	0.856857	-0.00746	0.010814
**Prefrontal**	**Temporal**	**Theta**	0.07831	-0.0145	0.005509	0.442816	-0.01267	0.006601	0.856857	0.007117	0.008048
**Prefrontal**	**Occipital**	**Theta**	0.950258	-0.00268	0.007823	0.824191	0.003928	0.008875	0.856857	-0.00531	0.011148
**Frontal**	**Central**	**Theta**	0.978715	0.001263	0.008379	0.824191	-0.00422	0.009319	0.856857	0.00608	0.010843
**Frontal**	**Parietal**	**Theta**	0.978715	0.000223	0.007444	0.824191	-0.00596	0.008279	0.856857	0.00902	0.009632
**Frontal**	**Temporal**	**Theta**	0.000799	-0.02209	0.005139	0.029129	-0.01687	0.005245	0.043646	0.019733	0.006402
**Frontal**	**Occipital**	**Theta**	0.265504	-0.01269	0.00646	0.737342	-0.00782	0.006858	0.856857	0.002248	0.008743
**Central**	**Parietal**	**Theta**	0.877838	-0.00404	0.007361	0.849995	0.002525	0.007477	0.856857	-0.00224	0.008789
**Central**	**Temporal**	**Theta**	0.978715	-0.00016	0.006095	0.824191	0.004258	0.00665	0.856857	-0.00243	0.007912
**Central**	**Occipital**	**Theta**	0.877838	-0.00472	0.007122	0.824191	-0.00367	0.007586	0.856857	0.003207	0.009302
**Parietal**	**Temporal**	**Theta**	0.634585	0.008407	0.006526	0.737342	0.008663	0.007219	0.856857	-0.00781	0.008555
**Parietal**	**Occipital**	**Theta**	0.877838	-0.00532	0.006479	0.98959	0.000444	0.006452	0.856857	0.007582	0.007672
**Temporal**	**Occipital**	**Theta**	0.713938	0.006952	0.006467	0.737342	-0.00704	0.006854	0.934457	0.000739	0.00896
**Prefrontal**	**Frontal**	**Alpha**	0.818634	0.004344	0.005468	0.864137	0.01043	0.006099	0.735594	-0.01007	0.007373

**Prefrontal**	**Central**	**Alpha**	0.89186	0.003563	0.013988	0.864137	0.007946	0.014894	0.805153	0.007071	0.01747
**Prefrontal**	**Parietal**	**Alpha**	0.818634	0.009521	0.014492	0.864137	-0.00413	0.016193	0.805153	0.011095	0.018856
**Prefrontal**	**Temporal**	**Alpha**	0.818634	-0.00935	0.010218	0.864137	-0.0053	0.011758	0.735594	0.015064	0.014253
**Prefrontal**	**Occipital**	**Alpha**	0.89186	0.003931	0.008755	0.864137	-0.00263	0.009961	0.735594	0.017911	0.012518
**Frontal**	**Central**	**Alpha**	0.89186	-0.00229	0.010773	0.864137	-0.00757	0.010915	0.735594	0.015948	0.01246
**Frontal**	**Parietal**	**Alpha**	0.818634	0.010132	0.011525	0.864137	0.003865	0.012229	0.805153	0.005512	0.014133
**Frontal**	**Temporal**	**Alpha**	0.89186	-0.00175	0.007895	0.864137	0.009658	0.008007	0.944456	-0.00068	0.009762
**Frontal**	**Occipital**	**Alpha**	0.964579	-0.00035	0.007859	0.864137	0.001441	0.008392	0.735594	0.01226	0.01071
**Central**	**Parietal**	**Alpha**	0.818634	0.005316	0.008318	0.864137	-0.00694	0.009316	0.785784	0.00812	0.011215
**Central**	**Temporal**	**Alpha**	0.818634	-0.00637	0.006336	0.864137	0.00829	0.006522	0.805153	-0.00328	0.007622
**Central**	**Occipital**	**Alpha**	0.818634	0.004125	0.006796	0.864137	-0.0017	0.007239	0.785784	0.007554	0.008877
**Parietal**	**Temporal**	**Alpha**	0.818634	-0.00768	0.007003	0.864137	0.002498	0.007538	0.735594	0.01337	0.008862
**Parietal**	**Occipital**	**Alpha**	0.818634	-0.00679	0.007797	0.864137	0.007158	0.00814	0.785784	0.007615	0.009777
**Temporal**	**Occipital**	**Alpha**	0.818634	-0.005	0.006504	0.864137	0.008271	0.006893	0.938487	-0.00141	0.009012
**Prefrontal**	**Frontal**	**Beta**	0.900351	-0.00084	0.003367	0.949148	0.000755	0.003688	0.725786	-0.00352	0.004447
**Prefrontal**	**Central**	**Beta**	0.900351	-0.0026	0.004251	0.949148	0.000588	0.004656	0.870216	-0.00274	0.005491
**Prefrontal**	**Parietal**	**Beta**	0.900351	-0.00072	0.004109	0.949148	-0.00178	0.004817	0.994898	-0.00093	0.005649
**Prefrontal**	**Temporal**	**Beta**	0.900351	0.000783	0.003012	0.876459	0.002998	0.003609	0.870216	-0.00208	0.0044
**Prefrontal**	**Occipital**	**Beta**	0.170347	-0.00809	0.003117	0.12249	-0.00936	0.003444	0.008311	0.015543	0.004308
**Frontal**	**Central**	**Beta**	0.900351	0.001828	0.003404	0.949148	0.000453	0.00373	0.994898	-0.00037	0.004324
**Frontal**	**Parietal**	**Beta**	0.900351	0.000516	0.004104	0.949148	-0.00274	0.004565	0.725786	0.004552	0.005311
**Frontal**	**Temporal**	**Beta**	0.900351	-0.00159	0.002574	0.949148	-0.00018	0.002778	0.725786	0.002688	0.003427
**Frontal**	**Occipital**	**Beta**	0.897784	-0.00358	0.003377	0.667839	-0.00403	0.003424	0.108181	0.010845	0.004334
**Central**	**Parietal**	**Beta**	0.900351	0.002842	0.00362	0.667839	-0.00437	0.003909	0.725786	0.004021	0.004659
**Central**	**Temporal**	**Beta**	0.897784	0.003429	0.003278	0.949148	0.000908	0.003576	0.994898	-2.73E-05	0.004255

**Central**	**Occipital**	**Beta**	0.900351	-0.00153	0.003943	0.949148	-0.00122	0.004199	0.725786	0.004441	0.00515
**Parietal**	**Temporal**	**Beta**	0.897784	0.004371	0.003805	0.667839	0.005113	0.004132	0.280628	-0.00947	0.00487
**Parietal**	**Occipital**	**Beta**	0.900351	0.001204	0.003658	0.615602	0.005617	0.0036	0.905372	-0.00151	0.004272
**Temporal**	**Occipital**	**Beta**	0.863959	0.004466	0.002804	0.615602	0.004845	0.00277	0.725786	-0.00451	0.003607
**Prefrontal**	**Frontal**	**Gamma**	0.86207	-0.00284	0.004801	0.95981	-0.00147	0.005389	0.613833	-0.01043	0.00652
**Prefrontal**	**Central**	**Gamma**	0.86207	0.002714	0.006351	0.95981	-0.00302	0.007204	0.613833	-0.00886	0.008558
**Prefrontal**	**Parietal**	**Gamma**	0.86207	-0.00287	0.006651	0.95981	-0.00343	0.007676	0.613833	-0.00814	0.008981
**Prefrontal**	**Temporal**	**Gamma**	0.86207	-0.00336	0.003545	0.95981	-0.0025	0.004103	0.675234	-0.00341	0.004977
**Prefrontal**	**Occipital**	**Gamma**	0.86207	-0.00403	0.003278	0.95981	-0.00147	0.003567	0.613833	-0.0041	0.004452
**Frontal**	**Central**	**Gamma**	0.86207	-0.00389	0.004401	0.95981	0.000248	0.004895	0.613833	-0.00597	0.005695
**Frontal**	**Parietal**	**Gamma**	0.86207	-0.00211	0.005422	0.95981	0.004014	0.00603	0.613833	-0.0072	0.007016
**Frontal**	**Temporal**	**Gamma**	0.86207	0.000483	0.002675	0.95981	0.002461	0.003013	0.613833	-0.00656	0.003752
**Frontal**	**Occipital**	**Gamma**	0.86207	-0.00418	0.003576	0.95981	0.000908	0.003785	0.908328	0.00123	0.004824
**Central**	**Parietal**	**Gamma**	0.86207	-0.0062	0.005082	0.95981	-0.00548	0.005488	0.688569	0.003922	0.006542
**Central**	**Temporal**	**Gamma**	0.86207	-0.00425	0.004441	0.95981	-0.00455	0.004789	0.908328	0.001094	0.005675
**Central**	**Occipital**	**Gamma**	0.86207	-0.00427	0.007332	0.95981	-0.00405	0.007518	0.613833	0.010692	0.009081
**Parietal**	**Temporal**	**Gamma**	0.86207	-0.00097	0.005555	0.95981	-0.00333	0.006146	0.95621	0.000401	0.007283
**Parietal**	**Occipital**	**Gamma**	0.86207	0.003256	0.011002	0.95981	-0.00202	0.011656	0.613833	0.012746	0.014047
**Temporal**	**Occipital**	**Gamma**	0.86207	-0.00142	0.003879	0.95981	0.000435	0.004111	0.675234	0.003798	0.005374
**Prefrontal**	**Frontal**	**Broad**	0.862537	-0.00111	0.002902	0.939314	-0.00107	0.003362	0.526778	-0.00516	0.004088
**Prefrontal**	**Central**	**Broad**	0.637866	0.004874	0.003874	0.653037	0.004979	0.004394	0.305583	-0.00994	0.00522
**Prefrontal**	**Parietal**	**Broad**	0.761795	0.004632	0.005031	0.920486	-0.00248	0.005899	0.869926	0.001665	0.006919
**Prefrontal**	**Temporal**	**Broad**	0.862537	0.000531	0.003054	0.920486	-0.00175	0.003659	0.869926	-0.00178	0.004462
**Prefrontal**	**Occipital**	**Broad**	0.842919	-0.00239	0.003963	0.920486	-0.00324	0.004509	0.869926	0.001071	0.005667
**Frontal**	**Central**	**Broad**	0.761795	0.002736	0.003273	0.967861	0.000306	0.003641	0.869926	-0.00209	0.004236

**Frontal**	**Parietal**	**Broad**	0.862537	0.001105	0.003882	0.967861	0.000175	0.004318	0.869926	0.000826	0.005024
**Frontal**	**Temporal**	**Broad**	0.761795	-0.00196	0.002341	0.653037	-0.00313	0.002637	0.817197	0.002573	0.003284
**Frontal**	**Occipital**	**Broad**	0.862537	-0.00073	0.003185	0.920486	-0.00195	0.003314	0.869926	0.000727	0.004212
**Central**	**Parietal**	**Broad**	0.842919	-0.00185	0.003166	0.920486	-0.00189	0.003559	0.451954	0.006234	0.004289
**Central**	**Temporal**	**Broad**	0.329421	0.006289	0.003121	0.374463	0.007709	0.003406	0.305583	-0.00872	0.004052
**Central**	**Occipital**	**Broad**	0.862537	-0.00116	0.003094	0.967861	0.000447	0.003295	0.817197	0.003209	0.004041
**Parietal**	**Temporal**	**Broad**	0.329421	0.006339	0.00339	0.518103	0.005628	0.00375	0.451954	-0.00661	0.004444
**Parietal**	**Occipital**	**Broad**	0.334181	0.006139	0.003561	0.518103	0.005864	0.003817	0.869926	-0.00151	0.004614
**Temporal**	**Occipital**	**Broad**	0.091586	0.007748	0.002751	0.374463	0.005633	0.00283	0.305583	-0.00729	0.003693

File:

File S1. Safety & Equine Welfare checklist (pre/during/post-session)

Pre-session (to be completed by equine handler/therapist): - Veterinary clearance current (Y/N) - Horse soundness check (lameness, appetite, wounds) (Y/N) - Tack fit & condition OK (Y/N) - Environmental check (footing, temperature, noise) (Y/N) - Horse rested: not exceeded 2 sessions prior day (Y/N)

During session (handler/therapist): - Observe equine stress signs (ears, tail, vocalization) — any observed? (Y/N) If Y, action taken: ________ - Monitor horse gait consistency (Y/N) - Ensure appropriate breaks & water access post-session (Y/N)

Post-session: - Cool-out and grooming completed (Y/N) - Horse fed/rehydrated as per protocol (Y/N) - Record any adverse events or changes in behavior (details): ________________

Handler signature: _________________________ Date: __________

Sample: Pre-session (completed by equine handler/therapist)

- Veterinary clearance current: **Y**
- Horse soundness check (lameness, appetite, wounds): **Y** (no lameness; good appetite)
- Tack fit & condition OK: **Y**
- Environmental check (arena footing acceptable; ambient temperature within acceptable range)
- Horse rested: not exceeded 2 sessions prior day: **Y** (H3 had one session previous day)

During session (handler/therapist)

- Observe equine stress signs (ears, tail, vocalization) — any observed? **N**
  If Y, action taken: —
- Monitor horse gait consistency: **Y** (regular walk rhythm)
- Ensure appropriate breaks & water access post-session: **Y**

Post-session

- Cool-out and grooming completed: **Y**
- Horse fed/rehydrated as per protocol: **Y**
- Record any adverse events or changes in behavior: **N**

Handler signature: HND1 — Date: StudyWeek3_Session5

(Notes: H3 substituted for H5 on this date due to vet check for H5; H3 showed normal gait and no stress signs.)

File S2. Fidelity checklist (use one per session)

Fidelity Checklist - Session record

- Date: __________
- Participant ID: __________
- Session #: ___ Start time: ____ End time: ____
- Primary horse ID: ________ Secondary (if substituted): ________
- Therapist: __________ Observer: __________

Checklist items (mark Y/N or value; comment if N / deviation): - Orientation & safety review completed (Y/N): __
- Helmet and tack checked (Y/N): **- Pre-session horse welfare check completed (Y/N):** - Ground-based preparatory activity performed (Y/N): **- Core mounted activities performed (Y/N):** - Core mounted duration (minutes): **- Social–emotional tasks embedded (Y/N):** - Cool-down/reflection completed (Y/N): **- Any deviations from manual (Y/N):** If yes, describe: ______________________ - Was substitution of horse/personnel needed? (Y/N): __ If yes, reason: ____________ - Participant tolerated session well (Y/N): __ Comments: _______________________

Observer signature: ____________________ Therapist signature: ___________________

1) Fidelity checklist (Sample: Session 1, Session 6 ‹Session 12)

Fidelity checklist - Session record (Sample A: Participant S01 - Session 1)

- Date: StudyWeek1_Session1
- Participant ID: S01
- Session #: 1 Start time: 09:10 End time: 09:35
- Primary horse ID: H1 Secondary (if substituted): —
- Therapist: T01 Observer: O01

Checklist items (Y/N; comments if N / deviation):

- Orientation & safety review completed: **Y**
- Helmet and tack checked: **Y**
- Pre-session horse welfare check completed: **Y**
- Ground-based preparatory activity performed: **Y**
- Core mounted activities performed: **Y**
- Core mounted duration (minutes): **23**
- Social–emotional tasks embedded: **Y** (turn-taking during grooming)

- Cool-down/reflection completed: **Y**
- Any deviations from manual: **N**
- Was substitution of horse/personnel needed?: **N**
- Participant tolerated session well: **Y**
- Comments: Participant required minimal verbal prompts for postural tasks; no signs of distress.

Observer signature: O01 (initials) — Therapist signature: T01 (initials)

2) Fidelity checklist - Sample B: Participant S07 - Session 7 (horse substitution logged)

- Date: StudyWeek3_Session6
- Participant ID: S07
- Session #: 6 Start time: 11:30 End time: 11:55
- Primary horse ID: H5 Secondary (if substituted): **H3** (substitution due to H5 veterinary check)
- Therapist: T01 Observer: O02

Checklist items:

- Orientation & safety review completed: **Y**
- Helmet and tack checked: **Y**
- Pre-session horse welfare check completed: **Y**
- Ground-based preparatory activity performed: **Y**
- Core mounted activities performed: **Y** (adapted)
- Core mounted duration (minutes): **18** *(adapted — reduced by 7 min due to participant fatigue)*
- Social–emotional tasks embedded: **Y**
- Cool-down/reflection completed: **Y**
- Any deviations from manual: **Y** — *Core mounted duration reduced; task 3 (single-leg stance) downgraded to assisted weight-shift.*
  Describe: Participant displayed mild fatigue; therapist shortened mounted phase and replaced higher-level balance task with supported weight-shifts. Documented in log.
- Was substitution of horse/personnel needed?: **Y** — *H5 substituted by H3 due to scheduled veterinary dental check.*
  If yes, reason: H5 unavailable (veterinary).
- Participant tolerated session well: **Y** (with adaptation)

- Comments: Substitution performed smoothly; H3 demonstrated similar gait characteristics; adaptation allowed safe completion.

Observer signature: O02 — Therapist signature: T01

3) Fidelity checklist - Sample C: Participant S12 - Session 12 (final assessment)

- Date: StudyWeek6_Session12
- Participant ID: S12
- Session #: 12 Start time: 14:00 End time: 14:30
- Primary horse ID: H2 Secondary: —
- Therapist: T01 Observer: O01

Checklist items:

- Orientation & safety review completed: **Y**
- Helmet and tack checked: **Y**
- Pre-session horse welfare check completed: **Y**
- Ground-based preparatory activity performed: **Y**
- Core mounted activities performed: **Y**
- Core mounted duration (minutes): **25**
- Social–emotional tasks embedded: **Y**
- Cool-down/reflection completed: **Y**
- Any deviations from manual: **N**
- Was substitution of horse/personnel needed?: **N**
- Participant tolerated session well: **Y**
- Comments: Final session completed as per manualised protocol; standard task battery performed for outcome comparison.

Observer signature: O01 — Therapist signature: T01

Figures:

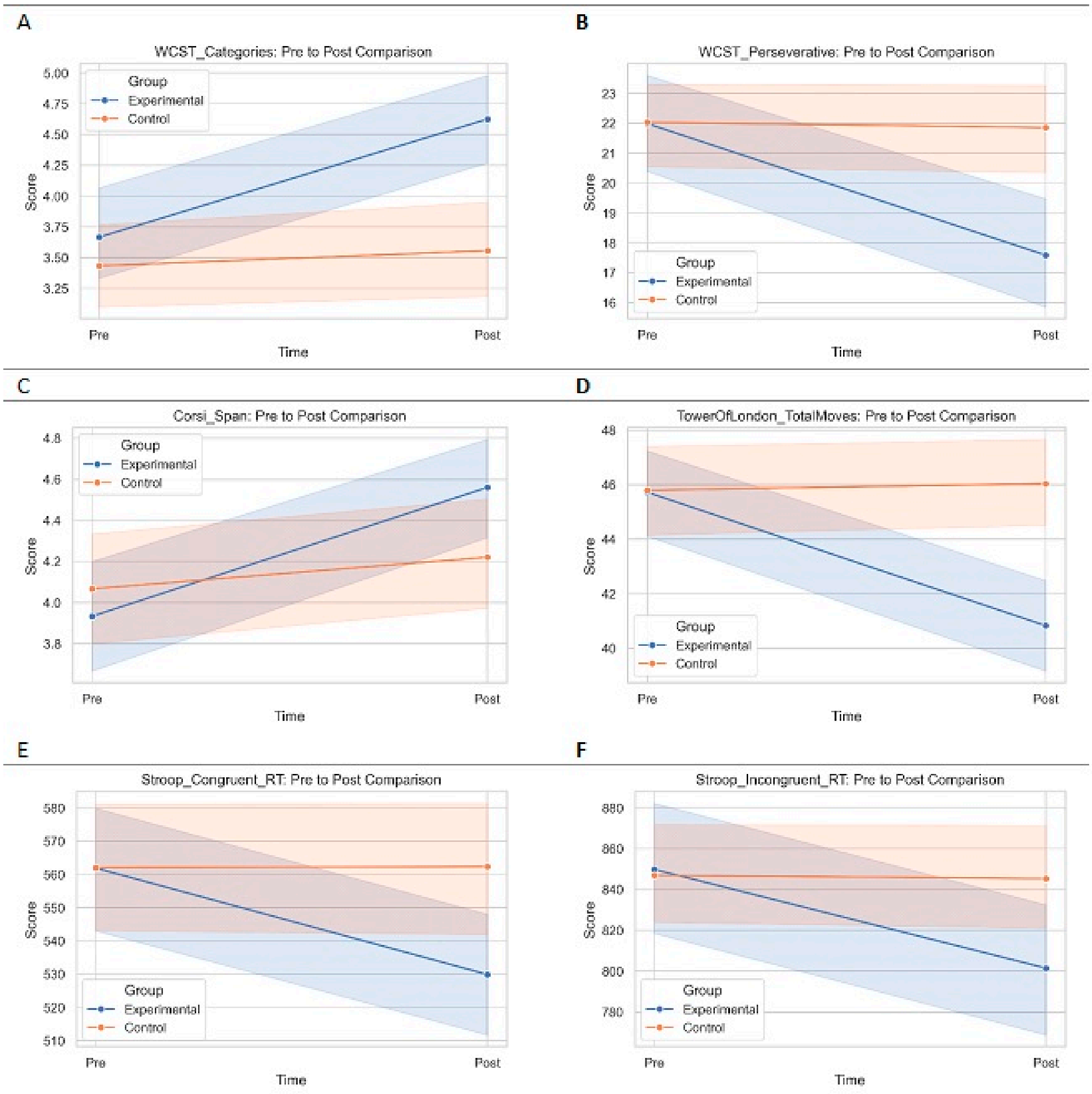

Figure S1. Group Differences in Cognitive Performance Pre- and Post-Intervention

Figure Legend. Figure S3 illustrates the mean cognitive task performance scores for the intervention (hippotherapy) and control groups at pre- and post-intervention assessments. The figure includes:

- **(A) WCST Category Completion:** Plot showing the relationship between pre- and post-intervention scores for WCST Category Completion; higher scores reflect better cognitive flexibility and set-shifting performance.
- **(B) WCST Perseverative Errors:** Plot showing the relationship between pre- and post-intervention scores for WCST Perseverative Errors; lower scores reflect improved executive control and reduced cognitive rigidity.
- **(C) Corsi Span Task Capacity:** Plot showing the relationship between pre- and post-intervention scores for Corsi Span Task Capacity; higher scores indicate superior visuospatial working memory.

- **(D) Tower of London Total Moves:** Plot showing the relationship between pre- and post-intervention scores for Tower of London Total Moves; lower scores indicate more efficient planning and problem-solving ability.
- **(E) Stroop Task Congruent Reaction Time (RT):** Plot showing the relationship between pre- and post-intervention scores for Stroop Congruent RT; lower RT denotes enhanced processing speed.
- **(F) Stroop Task Incongruent Reaction Time (RT):** Plot showing the relationship between pre- and post-intervention scores for Stroop Incongruent RT; lower RT suggests improved inhibitory control and executive functioning.

Overall, group-specific performance changes over time demonstrate a differential effect of hippotherapy on cognitive functioning, indicating potential cognitive benefits associated with the intervention...

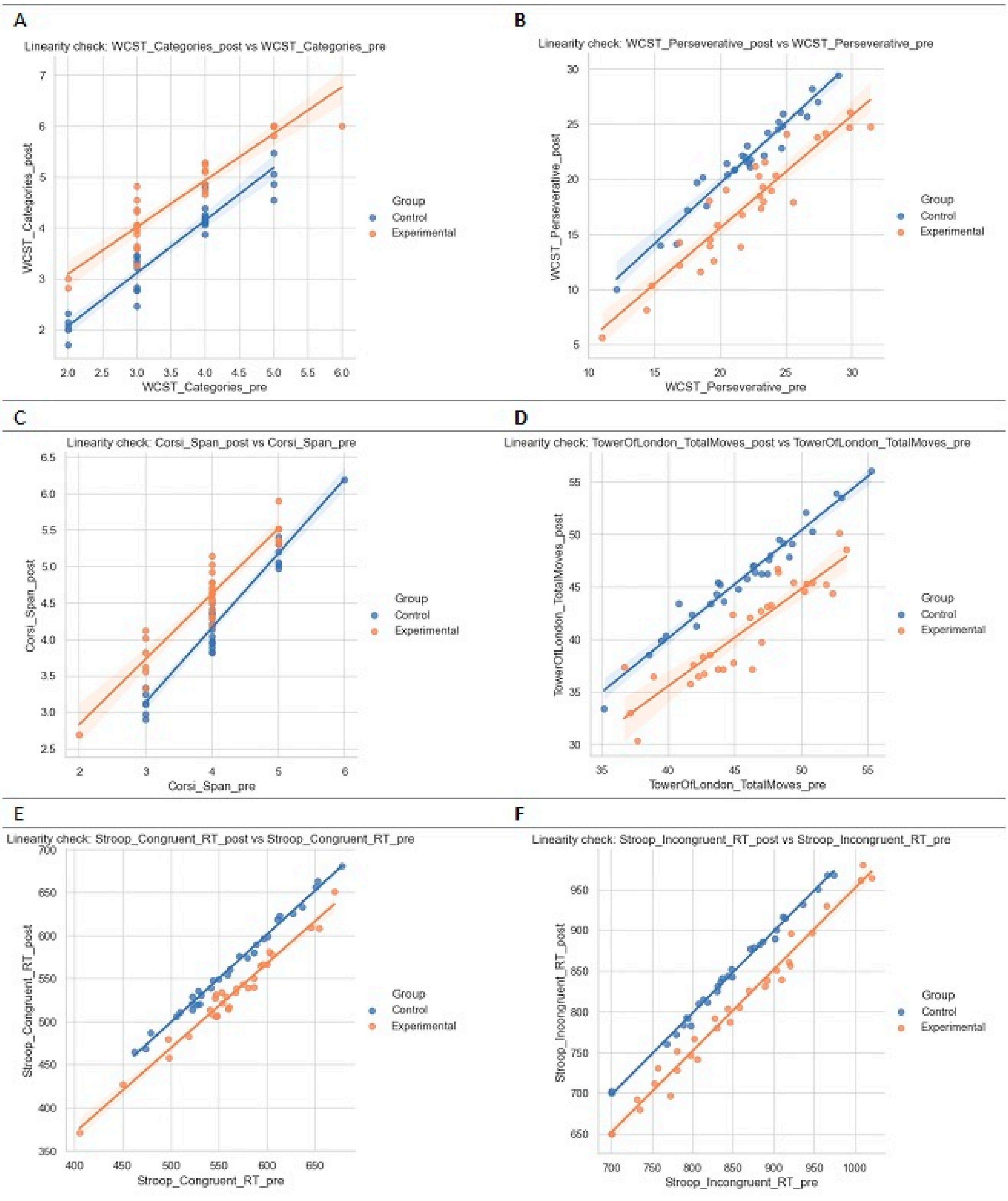

Figure S2. Linearity Assessment of Pre- to Post-Intervention Changes

Figure Legend. Figure S4 illustrates the linearity of the relationship between pre-intervention and post-intervention scores for key cognitive variables, assessed visually. Each panel is dedicated to a specific cognitive task:

- **(A) WCST Categories:** Plot showing the relationship between pre- and post-intervention scores for WCST Categories.
- **(B) WCST Perseverative Errors:** Plot showing the relationship between pre- and post-intervention scores for WCST Perseverative Errors.
- **(C) Corsi Span:** Plot showing the relationship between pre- and post-intervention scores for Corsi Span.
- **(D) Tower of London Total Moves:** Plot showing the relationship between pre- and post-intervention scores for Tower of London Total Moves.
- **(E) Stroop Congruent Reaction Time (RT):** Plot showing the relationship between pre- and post-intervention scores for Stroop Congruent RT.
- **(F) Stroop Incongruent RT:** Plot showing the relationship between pre- and post-intervention scores for Stroop Incongruent RT.

These plots are crucial for evaluating the assumption of linearity, a prerequisite for several statistical models used in analyzing change scores. Significant deviations from a linear pattern may suggest non-linear effects or signal potential issues in model specification, impacting the interpretation of effect sizes

---

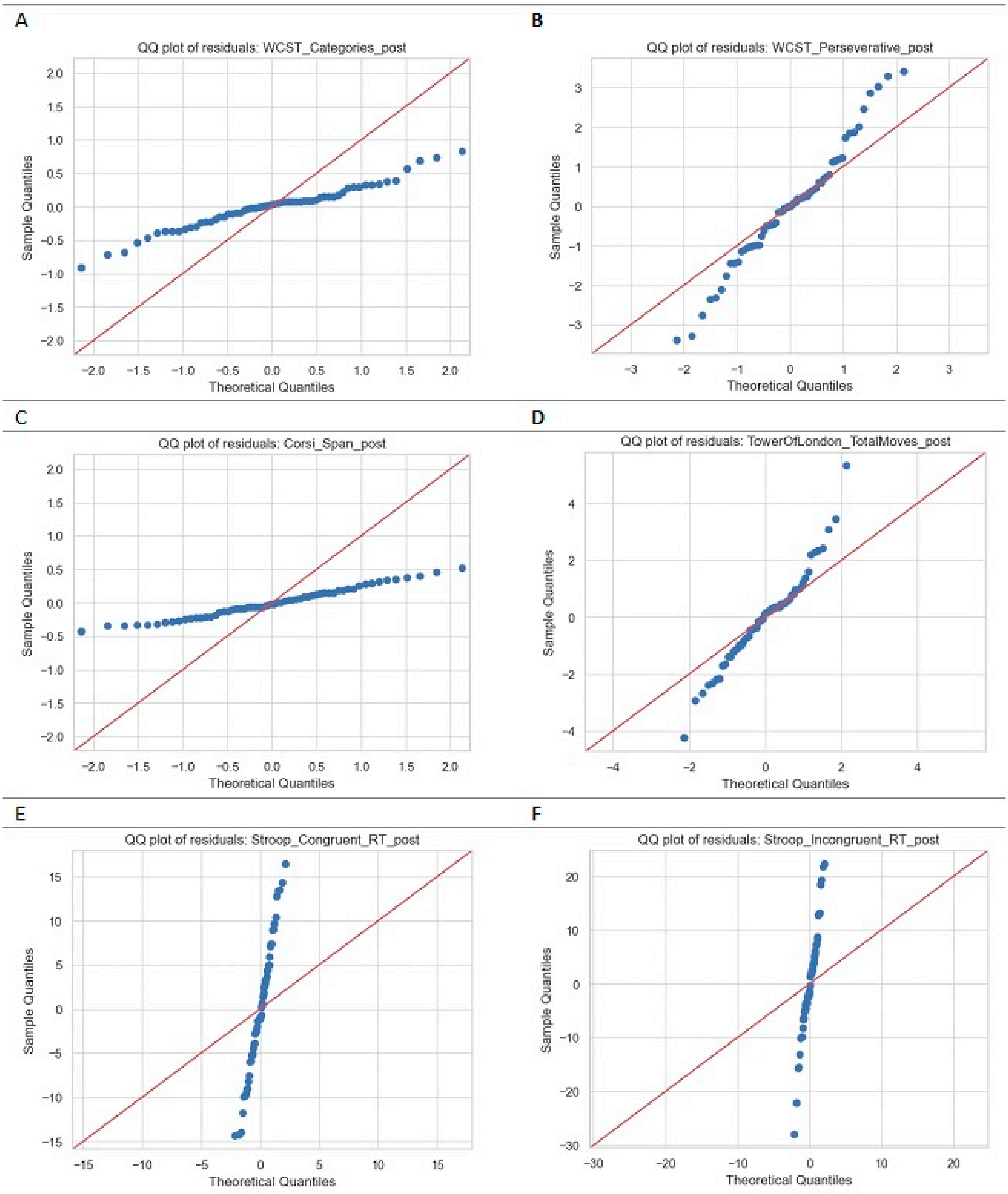

Figure S3. Normality Assessment of Model Residuals via QQ Plots

Figure Legend. Figure S5 displays Quantile-Quantile (QQ) plots used to assess the normality of residuals for statistical models examining pre- to post-intervention changes in key cognitive variables. Each panel corresponds to a specific cognitive task:

- **(A) WCST Categories:** QQ plot for residuals related to the WCST Categories variable.
- **(B) WCST Perseverative Errors:** QQ plot for residuals related to the WCST Perseverative Errors variable.
- **(C) Corsi Span:** QQ plot for residuals related to the Corsi Span variable.
- **(D) Tower of London Total Moves:** QQ plot for residuals related to the Tower of London Total Moves variable.
- **(E) Stroop Congruent Reaction Time (RT):** QQ plot for residuals related to the Stroop Congruent RT variable.
- **(F) Stroop Incongruent RT:** QQ plot for residuals related to the Stroop Incongruent RT variable.

The observed quantiles of the residuals are plotted against the theoretical quantiles of a standard normal distribution. A close alignment of data points along the diagonal reference line in each plot indicates that the model residuals approximate a normal distribution, thereby validating a key assumption for the reliability of the inferential statistics and effect size estimates.
